# Two-Stage Machine Learning for Thermo-Mechanical Properties Optimization of Carbon-Filled Polyimide Composites

**DOI:** 10.3390/ma19173667

**Published:** 2026-08-28

**Authors:** Yu Zhang, Wenting Zhao, Luling He, Qiong Li, Weibin Ma, Tongle Xu, Peng Ding

**Affiliations:** 1 Research Center of Nano Science and Technology, College of Sciences, Shanghai University, Shanghai 200444, China; 2 Department of Physics, College of Sciences, Shanghai University, Shanghai 200444, China; 3 Shanghai Key Laboratory of Atomic Control and Application of Inorganic 2D Supermaterials, Shanghai University, Shanghai 200444, China

**Keywords:** composites, machine learning, tensile strength, thermal conductivity

## Abstract

**Highlights:**

- A two-stage ML framework decouples processing and formulation optimization.
- The two-stage strategy allows concurrent improvement of TS and TC.
- The CF/SA ratio serves as a composition-derived descriptor for interfacial effects.
- Feature construction enhances model reliability under small-data constraints.

**Abstract:**

The rational design of high-performance carbon-filled polyimide (CF/PI) composites is challenged by complex interactions among processing parameters, composition, and interfacial effects. Here, we address thermo-mechanical co-optimization in CF/PI composites through two-stage processing and formulation design. An experimental dataset was employed to construct processing and composition datasets. A CatBoost model was developed to relate hot-pressing parameters to the tensile strength (TS) of pure PI, enabling inverse optimization of processing conditions, with quantitative experimental validation of the predicted processing windows (RMSE = 3.89 MPa). Within the optimized processing window, an XGBoost-based composition-property model was further established to perform high-throughput screening and multi-objective optimization of TS and thermal conductivity (TC). To quantitatively account for interfacial effects, the mass ratio of carbon-filled to sizing agent (CF/SA) was introduced as a composition-derived feature. Model interpretation reveals that CF and graphene positively contribute to TC but negatively affect TS. Notably, formulations with CF/SA > 2 tend to achieve a more favorable TS–TC balance. Experimental validation of model-selected composite formulations confirmed the predicted trends for both TS and TC. These findings suggest that interfacial regulation is a key lever for balancing mechanical strength and thermal transport in CF/PI composites.

## 1. Introduction

With the relentless move toward higher integration, power densities, and miniaturization, electronic devices accumulate heat rapidly within their confined envelopes, jeopardizing operational stability and shortening service life. Thermal conductivity (TC), mechanical robustness, and thermal stability become the defining attributes for reliable, long-term packaging materials. Among the available candidates, carbon-fiber-reinforced thermoplastic polyimide (CF/TPI) is widely recognized for its outstanding mechanical strength and inherently high TC, rendering it an attractive choice for high-reliability aerospace electronics [1,2,3]. In industrial practice, interfacial modifiers are among the most effective means of tailoring carbon-fiber surfaces [4,5,6]. Rational combinations of dissimilar fillers, such as CF/graphene (GP) or carbon-nanotube (CNT)/GP hybrids, further tune interfacial matching and create synergistic, percolated heat-conduction networks [4,7]. E. Ciecierska et al. demonstrated that reinforcing rigid PU foam with CNTs or graphite elevated flexural strength by ~20% while enhancing thermal, mechanical, and flame-retardant properties [8]. Liu et al. boosted composite TC by orienting fillers through carefully tuned processing parameters [9]. Performance gains stem not only from the intrinsic attributes of each constituent [10,11], but also from multi-scale interactions, filler–filler [12,13], filler–matrix [14] and additive–filler couplings. It is further constrained by the curing process and process-induced deformation [15]. Judicious selection of cure schedules, filler types, and loadings offers a credible route to simultaneous optimization of thermal and mechanical performance.

However, exploring this vast compositional-process design space experimentally is both time-consuming and costly [16]. The main challenge stems from the strong coupling among three groups of factors: (1) processing parameters (temperature, pressure, time), which govern multi-physical interactions including heat transfer, cure kinetics, and stress evolution [17]; (2) formulation variables (filler type, content, hybrid ratios), where synergistic or antagonistic effects among dissimilar fillers create highly nonlinear composition-property relationships; and (3) interfacial effects involving the sizing agent (SA), which bridges the filler and the matrix [18] but whose role may vary with processing conditions and formulation. Under such multi-factor coupling, physical models also face limitations. Semi-empirical models, such as Voigt and Reuss bounds [19], are derived from parameters that are difficult to measure and rarely reported. The accuracy of the approximation methods is relatively low [20]. High-fidelity finite-element modeling can, in principle, capture detailed microstructural features and multi-physics couplings [21,22,23]. Actually, realistic representation of CF surface is a major challenge. Many simulations use GP planes as CF surface, with few considering GP edges as done by Semoto and Yoshizawa [24,25]. In reality, the carbon fiber surface is far more complex and, even when simplified, must include both planes and edges. Incorporating such realistic surface features, along with multiple fillers, interfacial layers, and processing conditions, demands prohibitive computational resources [26,27].

Machine learning (ML)-based materials research has demonstrated the ability to exceed the limitations of traditional physical modeling, thus establishing a new framework for effective materials design [28,29,30]. By analyzing patterns in the relationship between composite composition and properties, ML can help identify the optimal ratio of reinforcement to matrix to maximize tensile strength, thermal conductivity, or other desired properties, while minimizing weight [31]. Han et al. [32] used support-vector regression, Gaussian-process regression, and convolutional neural networks to predict the TC of porous materials with accuracy exceeding empirical formulae. Xu et al. [33] used a genetic-algorithm-optimized back-propagation network to model the processing-property relationship in thermoplastic vulcanizates, while Struzziero and Teuwen [34] employed multi-objective optimization to shorten cure cycles and temperature overshoot in VARTM. Recent studies have specifically addressed the challenge of small experimental datasets in composite ML. Lai et al. [35] combined transfer learning with convolutional neural networks to predict composite strength from the data from 31 samples, achieving R^2^ up to 0.94 on unseen samples. Tran et al. [36] demonstrated that on a small dataset of only 65 samples, an ensemble of SVR and XGBoost with proper hyperparameter tuning can robustly predict tensile properties of hybrid polypropylene composites (R^2^ = 0.907 for strength, 0.881 for modulus), without the need for transfer learning or large-scale data. This highlights that careful model design alone can achieve reliable predictions under small-sample conditions.

Despite these successes, two major limitations persist in the context of CF/PI composites. First, conflicts between multiple properties remain a major challenge. TS of unfilled PI films depends on hot-pressing conditions, while their TC is governed primarily by the imidization and casting stages. In filled composites, TC improves with the formation of continuous filler networks that reduce phonon scattering, but excessive fillers can disrupt matrix integrity, introduce interfacial defects, and reduce tensile strength (TS). This disparity allows us to first optimize processing conditions for TS (Stage I), then focus on filler formulation to balance TS and TC (Stage II). Secondly, current models generally lack an effective composition-derived descriptor. Hassinger et al. [37] demonstrated that interfacial energy, quantified by descriptors such as the W_PF/W_FF adhesion ratio (the ratio of the work of adhesion between filler and polymer to the work of adhesion between filler to filler), governs nanoparticle dispersion more strongly than processing conditions, yet such descriptors require separate experimental measurements, making them unsuitable for high-throughput screening. Bao et al. [38] combined core-shell filler engineering with machine learning to optimize ZnO/PP composites. Their results showed that interfacial engineering played a decisive role in reinforcement and enabled quantitative links between filler structure and mechanical performance. Song et al. [39] made progress by digitally characterizing interfacial factors through multiscale microstructural parameters (e.g., skin-core structure, pore characteristics, graphitization degree) and establishing nonlinear mappings to macroscopic mechanical properties using artificial neural networks. A quantitative indicator for the complex interphase governed by SA remains elusive. While SA forms a confined interlayer that enhances crosslinking density and optimizes overall performance [1], its effect is deeply coupled with factors such as graphene content and hybrid filler ratios. Therefore, transforming this sizing agent-modulated interface from an isolated factor into a quantifiable variable within a multi-parameter system is essential for moving beyond empirical trial-and-error toward more rational composite design.

**Figure 1:**
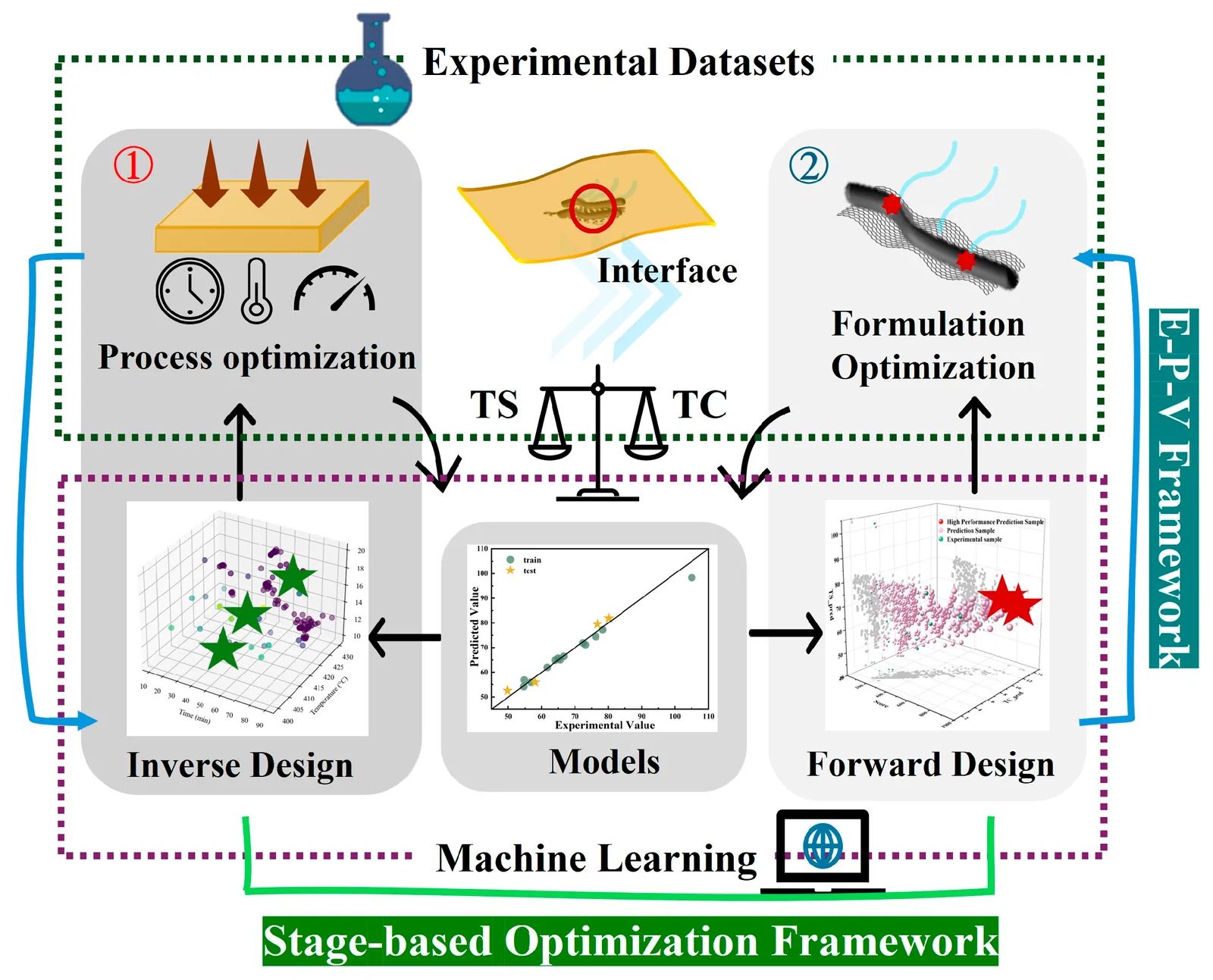
Two-stage ML framework for CF/PI composites, consisting of Stage I (processing optimization for pure PI resin) and Stage II (formulation optimization for CF/PI composites), following an experiment-prediction-validation (E-P-V) [40] logic.

To address the performance trade-offs in PI composites, this study proposes a ML-driven hierarchical design framework for CF/PI composites (Figure 1), following an “experiment-prediction-validation” (E-P-V) approach. Two datasets were constructed through experiments. In Stage I (processing optimization), recognizing that TC of unfilled PI is insensitive to processing, we focus solely on TS. A dataset of 140 pure PI films is used to train a CatBoost model, incorporating polynomial feature expansion to capture nonlinear process-property relationships. Bayesian optimization then inversely maps target TS values (80–110 MPa) to optimal hot-pressing parameters. In Stage II, using the optimal processing window from Stage I, we fabricate 20 CF/PI composite films with varying filler contents. Beyond using individual composition features, the CF/SA ratio was introduced as a composition-derived feature to reflect interface-related effects. An XGBoost model is then built to predict both TS and TC, and SHAP analysis reveals the contribution of CF/SA relative to other features. Experimental validation showed that appropriate SA regulation can contribute to simultaneous improvements in thermal transport and tensile performance. This data-driven framework effectively guides the design of CF/PI composites under complex processing constraints.

## 2. Materials and Methods

### 2.1. Experimental Synthesis and Validation

Authentic experimental datasets are crucial for enhancing the generalization capability, practicality, and robustness of machine learning models [41]. The “experiment-prediction-validation” framework can effectively advance the development of PI (PD450, Tianjin Geqite New Materials, Tianjin, China) composites [40]. Figure S1a shows a series of PI films and carbon-filled PI composite films designed and prepared via melt hot-pressing. Hot-pressing process parameters significantly influence the properties of polymer matrix composites. First, four hot-pressing conditions were determined in the PI synthesis experiments to optimize TS: hot-pressing temperature (400–430 °C), hot-pressing time (10–60 min), hot-pressing pressure (5–20 MPa), and film thickness (10–900 μm). Excessive thermal exposure (beyond 430 °C or >60 min) might induce over-crosslinking or chain degradation that deteriorates mechanical properties. Second, composite formulation parameters (CF (TYMF, Hunan Dongying Carbon Materials Technology Co., Ltd., Changsha, China) and GP (KNG-180-3, Xiamen Kaina Graphene Technology Co., Ltd., Xiamen, China) filler content 0–13.3 wt%, SA (TX-SAI-S01, Ningbo Institute of Materials Technology and Engineering, Chinese Academy of Sciences, Ningbo, China) content 0–5 wt%) were adjusted to construct phonon transport pathways, enhancing TC while maintaining TS. The selected composition ranges were determined based on our group’s preliminary experiments [1]. During blending (Figure S1b), a dual-mode interface layer formed via “covalent bonding-physical adsorption.” Oxygen-containing groups on the carbon filler surface react chemically with the SA, altering their surface properties. Part of SA is chemically bonded to the carbon filler surface, while the remainder adheres via physical adsorption [6]. During hot pressing, SA applied to the carbon filler surface permeates and crosslinks with the resin matrix. Experimental materials and characterization are summarized in Tables S1 and S2.

### 2.2. Datasets and Feature Engineering (FE)

Two datasets were used in this study, 140 samples/data points for pure PI films, with TS as the target and hot-pressing parameters (temperature, pressure, time, and thickness) as input features. Preliminary feature selection was performed based on Pearson correlation coefficients. The data were randomly split into training and test sets at a 4:1 ratio. The pure PI dataset was analyzed in three stages: the full dataset, a subset with outliers removed (based on the interquartile range method), and a subset with the thickness feature excluded. To capture nonlinear relationships and interaction effects, feature expansion was applied to the training set using the Polynomial Features (PF) from scikit-learn (version 1.5.1) (quadratic order with interaction terms), generating a feature space that includes original, squared, and pairwise interaction terms. Recursive Feature Elimination (RFE) was performed with embedded models including Ridge, Lasso, CatBoost, Partial Least Squares (PLS), and ElasticNet for feature selection. Automated FE was also compared using featuretools (relational feature synthesis) and autofeat (symbolic regression-driven generation), with Ridge regression used for modeling. Tree-based models such as decision trees and XGBoost provided interpretable rule-based outputs, while symbolic regression discovered relationships among features without manual polynomial construction. The second dataset, comprising 20 composite PI films with various fillers (e.g., CF and GP), included filler content as base features. Four additional features representing interfacial effects were introduced as ratios (e.g., CF/SA and GP/SA). The FE strategies developed for the pure matrix model (model-PI) were transferred and applied to the composite model (model-Co).

### 2.3. Machine Learning for Modeling

Based on the selected optimal feature subsets, further modeling was carried out. The CatBoost algorithm was used to construct the best model (model-PI) for the PI matrix. A Gaussian Process-based Bayesian optimization method (GPB) was applied for the inverse design of three processing parameters: time, temperature, and pressure. With model-PI as the performance predictor, the objective function was defined as the squared error between the predicted and target values. Through 200 iterations, the algorithm updated candidate parameter combinations and identified the optimal hot-pressing conditions corresponding to the minimum objective value. The optimal processing parameters derived from the inverse design with model-PI were transferred to the composite processing stage. XGBoost was employed to model the relationship between filler content and composite performance (model-Co-TS and model-Co-TC). A constrained compositional space was constructed in which the sum of PI, CF powder, and GP contents equals 1. Model-Cos were used to predict TS and TC for high-throughput combination. Due to the limited size of the composite dataset, Leave-One-Out Cross-Validation (LOO CV) and held-out testing were used jointly to assess model stability. The product of TS and TC was used as a scoring function to rank high-performance formulations satisfying dual-property requirements. In addition, Pareto-front analysis was performed to evaluate optimal trade-offs between TS and TC, supporting decision-making for experimental design. The Pareto-optimal solutions were identified using the NSGA-II algorithm (S2.5). To enhance model interpretability, SHAP was applied to provide global and local explanations of feature contributions and their positive or negative effects. All activities were conducted using Python (version 3.12), and model performance was evaluated using RMSE and R^2^ metrics.

## 3. Results and Discussion

### 3.1. Model-PI for Inverse Design (Stage I)

As a low-thermal-conductivity material (~0.22 W·m^−1^·K^−1^) [42], PI cannot meet the growing demands of thermal interface materials in microelectronics. Its TC is less sensitive to hot-pressing parameters compared to its mechanical performance. The TC of unfilled PI is governed primarily by its intrinsic chain packing density, which is largely determined by imidization chemistry rather than post-curing hot-pressing conditions [43]. Researchers at Tianjin Polytechnic University [44] have regulated the thermal conduction network of PI composites via electrospinning and hot pressing, yet the improvements in TC were mainly attributed to the addition of fillers (e.g., boron nitride, silver nanowires) rather than the PI matrix itself. Therefore, in Stage I, the influence of hot-pressing conditions was evaluated with respect to TS. A systematic experimental design based on the same PI was conducted, yielding a dataset of 140 entries under varying hot-pressing conditions.

**Figure 2:**
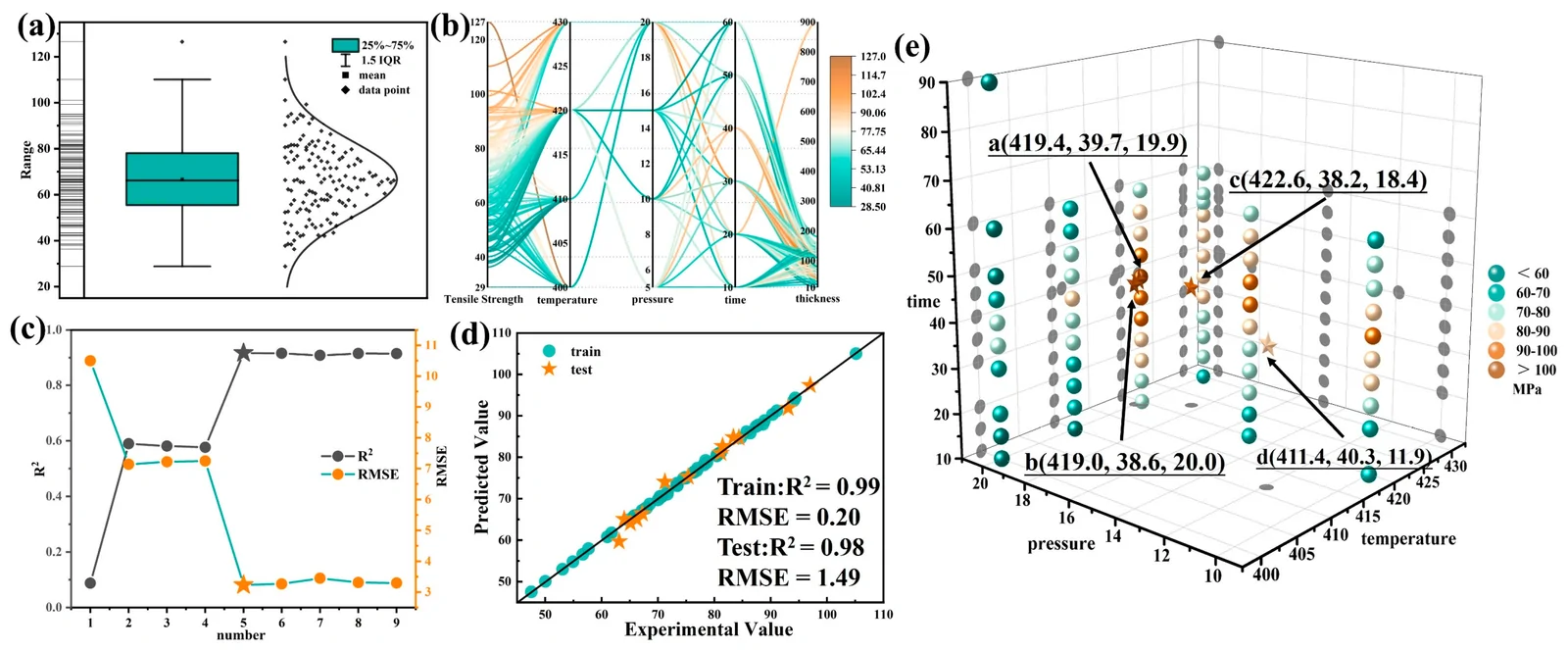
(**a**) Boxplot and distribution of experimental TS values for 140 PI films. (**b**) Parallel coordinates plot of processing parameters and TS. (**c**) The changes in R^2^ and RMSE for model-PI (10-fold-CV) built using RFE nested CatBoost with different numbers of descriptors were accurately described. (**d**) Scatter plots of the best model to predict the TS values versus their experimental values for the train and test sets. (**e**) 3D visualization of the four inverse design points within the framework of the original dataset.

Data visualization is an essential first step. Figure 2a presents the normal distribution curve and boxplot of TS, showing that most PI samples fall within the 60–80 MPa range. In Figure 2b, higher TS values tend to correlate with high-temperature and high-pressure conditions. From a polymer physics perspective, the nonlinear behavior of TS on temperature and time observed in Figure 2b arises from competing mechanisms: elevated temperatures (415–425 °C) and moderate holding times (30–50 min) promote chain mobility and crosslinking for optimal strength. The film thickness was controlled within 10–200 μm, and the effect of film thickness appears less pronounced. The heatmap in Figure S2a reveals weak correlations both between features and performance and among the features themselves.

Feature generation was applied to enrich the feature space, and nonlinear algorithms were considered more appropriate for modeling. Accurate TS prediction is critical for optimizing the production process and improving manufacturing efficiency, which requires building robust models with strong generalization [45]. Eleven methods were compared to identify the optimal descriptor set, with results summarized in Table S3. Symbolic regression performed the worst (test set R^2^ < 0.10), while linear models such as Ridge, Lasso, and PLS achieved test R^2^ values below 0.45, confirming the strong nonlinearity of the process-property relationship. PF-RFE-CatBoost achieved the best performance (R^2^ = 0.93 for training, R^2^ = 0.75 for test) but exhibited signs of overfitting. SHAP analysis (Figure S2b) indicated that temperature (+7.31) and time (+6.65) made significantly greater contributions to TS prediction than thickness (+2.91). Notably, a systematic study on PI films has shown that within the 50–200 μm range, TS varies by only ~9% (from 165 to 150 MPa) [46], confirming that thickness plays a secondary role compared to temperature and time. This finding aligns with the energy input mechanism of hot pressing, where temperature and time jointly govern the diffusion and rearrangement of molecular chains [47]. Outlier removal significantly improved the robustness and generalization of most models, as shown in Figure S3 and Table S4, although overfitting remained an issue for RFE-CatBoost. Standard testing for film TS, such as ASTM D882, has shown the influence of environmental factors (e.g., temperature and humidity) on test results [48], but thickness is typically not treated as an independent control factor, suggesting its marginal role. Thickness may influence TS indirectly through interaction terms (e.g., pressure * thickness), but its standalone contribution was insufficient to offset the noise introduced.

Based on feature importance analysis and domain knowledge, the thickness parameter was removed. As shown in Figure 2c and Table S5, the model’s R^2^ improved and stabilized progressively. Guided by the Occam’s razor principle [49], a subset of five selected descriptors was identified as the optimal feature set (Table S6). The CatBoost algorithm was selected for modeling, with hyperparameters optimized using the grid search method, as shown in Table S7. To ensure robustness, the dataset was randomly split ten times. As shown in Table S8, the model exhibited average R^2^ of 0.90 and RMSE of 3.01 MPa on the test sets, with the best split achieving R^2^ of 0.98 and RMSE of 1.49 MPa (Figure 2d), representing improvements of 92% and 80% compared to the initial model, respectively.

Based on the optimized model, an inverse design approach was proposed to enable efficient prediction in complex material discovery and design tasks [50]. The input is the target property value, and the output is the values of feature parameters. The model inversely maps target TS (80–110 MPa) to the processing parameter space. Figure S4 confirms convergence for four target values, while Figure S5 demonstrates the effectiveness and high success rate of the optimization process. Figure S6a–d presents the parameter importance plots during the optimization process for four experimental samples. The top and right parameter importance plots show the partial dependence of each parameter on the target value. Notably, temperature exhibits a monotonic decreasing trend, indicating that higher temperatures are generally beneficial for improving TS. Time shows a nonlinear U-shaped relationship, suggesting the existence of an optimal time point. Pressure has a relatively smaller but non-negligible effect, particularly under high target values. The two-dimensional contour maps visualize the interactions between processing parameters. Red stars represent the optimal parameter sets identified via Bayesian optimization, while black dots mark the sampled combinations evaluated during the search. This target-dependent variation arises because lower TS targets (e.g., 80 MPa) correspond to broader feasible regions in the processing parameter space, where many combinations of temperature, time, and pressure can achieve the goal. In contrast, higher TS targets (e.g., 110 MPa) require narrowly constrained extreme conditions (high temperature, moderate time, high pressure) to approach the material’s intrinsic strength limit.

As shown in Figure S7, the 3D parameter space reveals that both trends and optimal regions vary across different target values, reflecting the complex nonlinear interactions between processing parameters and material performance. The minimum objective value represents the squared error between the predicted property and the target value during the optimization process (Table S9). A lower objective value indicates a solution closer to the design target. Within the broad design space (pressure: 10–20 MPa; time: 10–90 min; temperature: 400–430 °C), lower target strengths yield smaller objective values and wider feasible regions, as blue spheres in Figure 2e. For higher target strengths, the feasible region narrows (pressure: 15–20 MPa; time: 35–45 min; temperature: 415–425 °C), and the objective values become lower. Precise matching regions during inverse design is important.

### 3.2. Model Interpretability Analysis and Optimal Processing Validation

**Figure 3:**
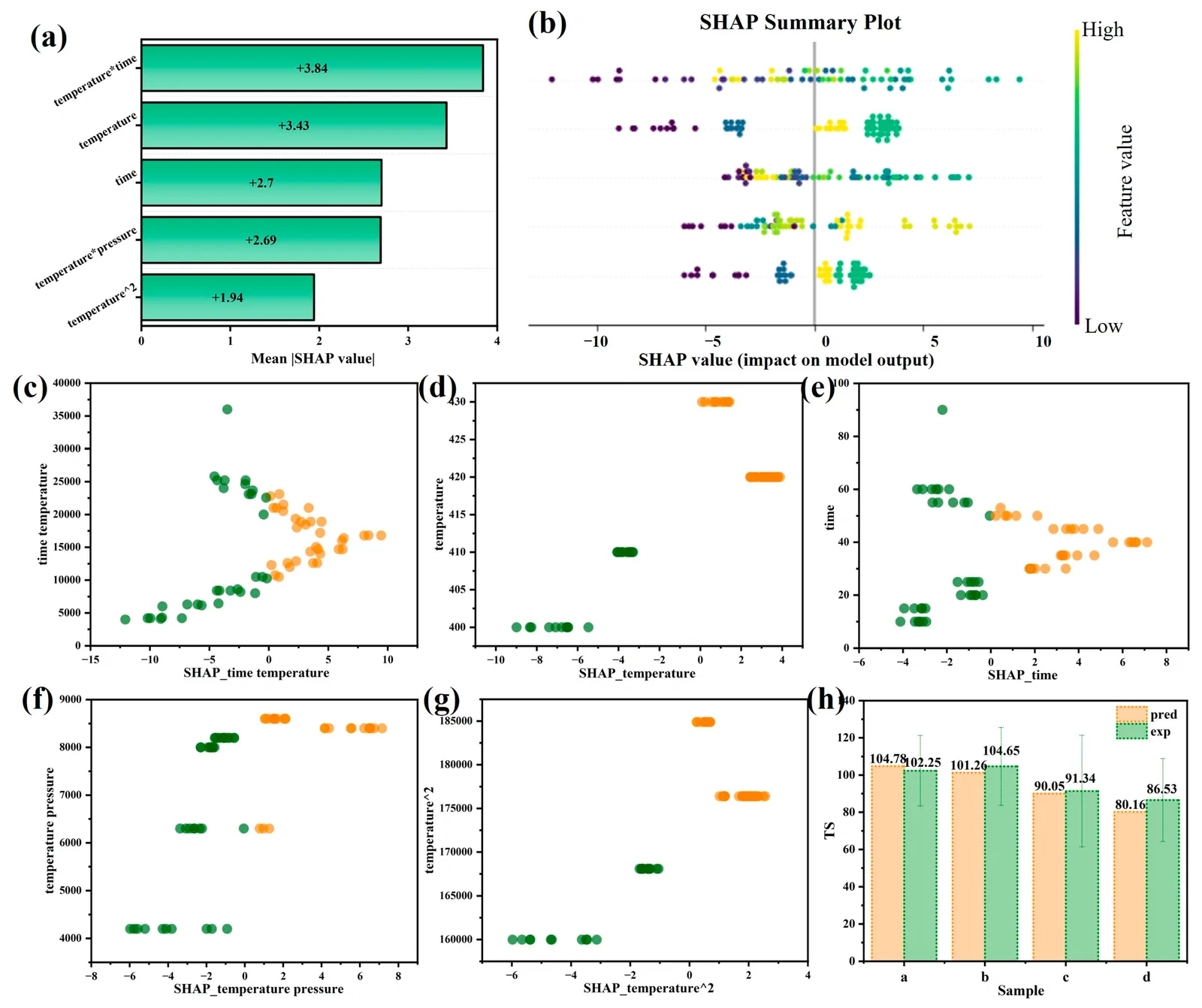
(**a**) The importance of ranking and (**b**) correlation of the best-chosen subset of the SHAP nested CatBoost algorithm. The relationship between the descriptor values and the SHAP values output for (**c**) time*temperature, (**d**) temperature, (**e**) time, (**f**) temperature*pressure, and (**g**) temperature^2. (**h**) Comparison plots of experimental and predicted values for the four candidate samples.

To further explore the relationship between processing parameters and PI’s TS and to provide guidance for experiments, we employed SHAP combined with domain knowledge to interpret the meaning of the selected descriptors. This method quantifies the “profit value” [51] of each input feature, reflecting how it contributes to the model in cooperation with other features. Feature contributions are measured by the average absolute impact of each feature on the target variable. Figure 3a and Figure 3b display the feature importance rankings and SHAP dependence of features. The interaction term time*temperature was the most influential descriptor, with optimal values falling in the range of 1*10^4^ to 2*10^4^ (Figure 3c). Temperature (Figure 3d) showed a positive contribution, with performance potentially improving above 420 °C. This can be attributed to the increased molecular mobility, improved flowability, and reduced viscosity of the polymer at elevated temperatures [52]. However, further increasing the temperature beyond this range may introduce thermal degradation risks, so the processing window should be selected cautiously. Time exhibited a nonlinear effect (Figure 3e), with an optimal range of 30–50 min. Insufficient hot-pressing time may result in incomplete curing or crosslinking, compromising structural integrity. Excessive time may lead to thermal degradation or over-crosslinking of components, ultimately deteriorating composite performance. These results further support the rationality of the high-performance targets boundaries established during the inverse design process. Additionally, both temperature*pressure (Figure 3f) and temperature^2 (Figure 3g) showed positive contributions to TS, whereas pressure had minimal effect. These trends are consistent with those reported by Yang et al. [52] for PEEK during hot pressing, regarding the roles of temperature, time, and pressure.

Figure 2e illustrates the positions of the four inverse-designed experimental samples within the original dataset. Table 1 and Figure 3h show that the absolute errors (Δ) between the experimental TS (TS^exp^) and the target output (TS^output^) ranged from 1.29 to 6.37 MPa. The model demonstrated good accuracy (Δ < 3.5 MPa) within the 90–100 MPa range, and deviations were observed near the boundary of the dataset. When the input target (TS^input^) approached the upper bound of the dataset (105.12 MPa), the model output (TS^output^) stayed near this limit instead of aligning with the input value (104.78 < 110), and the experimental value (TS^exp^) was lower than the predicted one (102.25 < 104.78). This suggests that when the target performance exceeds the training set range, the model tends to suggest extreme process parameters, but the actual performance is constrained by the intrinsic strength limits of the material. Although 80 MPa samples were well represented in the dataset (Figure S6a), the specific processing conditions of sample-a revealed a prediction blind spot in the low-pressure, low-temperature, long-time region (Figure 2e). Overall, the four experimental samples yielded an RMSE of 3.89 MPa, validating the effectiveness of model in inverse design.

### 3.3. Model-Co for Forward Design (Stage II)

By optimizing hot-pressing parameters, the molecular alignment and compact structure of PI chains were improved, laying a solid foundation for both TS and TC. The introduction of stable SAs (which form interfacial gradient layers), CF, and GP fillers further enhanced the continuity of TC pathways and the efficiency of stress transfer through controlled adjustment of their contents. Based on the optimal hot-pressing conditions for PI films, the formulation of composite materials was further optimized. TS of the composites showed a right-skewed distribution, with most samples concentrated in the 60–100 MPa range, and high-strength samples (>100 MPa) accounting for 15% (Figure S8). Figure S9 revealed a complex interaction network among different fillers, with TS and TC forming a trade-off relationship due to opposing mechanisms: filler networks enhance phonon transport for TC but introduce interfacial thermal resistance and stress concentration sites that degrade TS [1]. TS was positively correlated with the matrix content but negatively correlated with the contents of fillers and SAs, while TC showed the opposite trend. Mechanical properties benefited from improved interfacial bonding, whereas TC was primarily governed by the role of fillers in network formation.

The same preprocessing and FE were applied to the composite dataset. Table S10 presents the comparison results of various modeling methods. Linear models (Ridge, ElasticNet) performed poorly due to the nonlinear data, whereas RFE-XGBoost emerged as the best-performing algorithm. Simpler features are more suitable for small datasets. Thus, modeling was conducted using four features based on filler content. As shown in Figure S10a, model performance improved with the number of features, R^2^ increased, and RMSE decreased, but optimal performance was achieved with three to four key features. SHAP analysis (Figure S10b) indicated that the important scores of SA-contents and GP-content were comparable, supporting the inclusion of both. Based on feature importance and the fixed fillers, a subset of four optimal features was selected. Figure S10c shows the plots of the experimental TS values that match the predicted *TS* values. The XGBoost model achieved an R^2^ of 0.98 and an RMSE of 2.29 on the training set, demonstrating high accuracy. It also performed well on the independent test set (R^2^ = 0.96, RMSE = 2.39), confirming its generalization capability. The same XGBoost method was applied to the TC model (Figure S10d).

**Figure 4:**
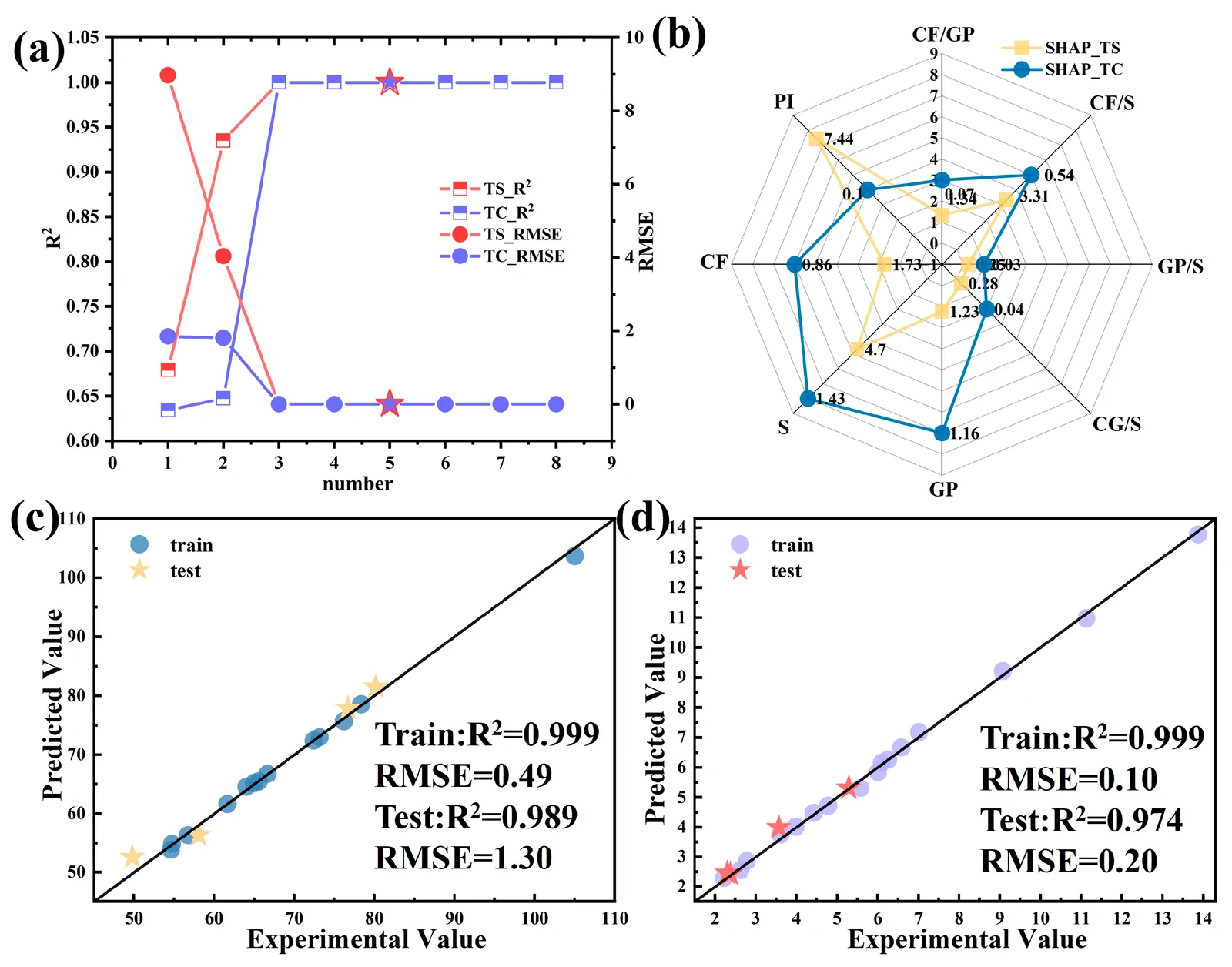
(**a**) The changes in R^2^ and RMSE for model-Co-TS (LOO-CV), built using RFE nested XGBoost with different numbers of descriptors, were accurately described. (**b**) Radar chart of eight features. Scatter plots of the best model to predict. (**c**) TS and (**d**) TC values versus their experimental values for the train and test sets.

The interfacial bonding between fillers and the matrix, as well as the physical and chemical interactions between filler surfaces and SA, influence composite performance [1]. Therefore, the interactions among fillers and the role of SA were expressed in the model as filler ratios and filler-to-SA ratios. Both FE and modeling continued using the XGBoost algorithm. FE results in Figure 4a show that although model performance for both TC and TS improved starting from three features, the rankings in Tables S11 and S12 consistently identified CF and CF/SA as key features. SHAP analysis (Figure 4b) further confirmed the importance of CF/SA, while SA-content and PI-content were particularly influential for TC and TS, respectively. Considering the combined influence on both properties, five features were ultimately selected, without compromising model performance. The hyperparameter optimization results are shown in Table S7. To mitigate overfitting risks given the limited dataset size, XGBoost models were regularized via shallow trees (max_depth = 3–6), L2 regularization (reg_lambda = 1–3), feature subsampling (colsample_bytree = 0.8), and early stopping. The final models, model-Co-TS and model-Co-TC, were established.

Compared with earlier models that did not consider filler–filler and filler–SA interactions, Figure 4c and Figure 4d demonstrate that model-Co-TS and model-Co-TC achieved significantly higher accuracy, with both R^2^ values reaching 0.999 and RMSEs of 0.49 and 0.10, respectively. The average RMSE of the test sets obtained by randomly partitioning the two datasets three times are 1.31 and 0.93, respectively. It is acknowledged that the high training R^2^ (0.999) may suggest potential overfitting given the limited sample size. Nevertheless, multiple safeguards were implemented to mitigate this risk, including shallow trees, L2 regularization, feature subsampling, and early stopping. The test RMSE values remain low relative to the property ranges (TS: 60–100 MPa, TC: 2–14 W·m^−1^·K^−1^). The two models thus exhibit practically useful generalization performance, highlighting the critical role of filler-SA interactions. Ma et al. [53] constructed the XGBoost model based on T, pH, and reactant to predict caproic acid production. The R^2^ values of their training and test sets were 0.998 and 0.885, respectively. Deng et al. [54] developed a symbolic regression-based model for optimizing rubber composite formulations, achieving R^2^ values of 0.97 and 0.95 for training and test sets, respectively. In comparison, our two models improved predictive consistency within this small-data regime, and the model predictions were further validated by experiments (Table 2).

To improve the model’s accessibility, an interactive online prediction tool was developed. As illustrated in Figure S11, researchers can easily leverage this toolkit by inputting target TS values and obtaining the corresponding hot-pressing conditions for PI or entering filler compositions to predict the TS and TC of composites (available at https://com-app.streamlit.app/, accessed on 25 August 2026).

### 3.4. Performance-Driven Composition Optimization and Experimental Comparison

**Figure 5:**
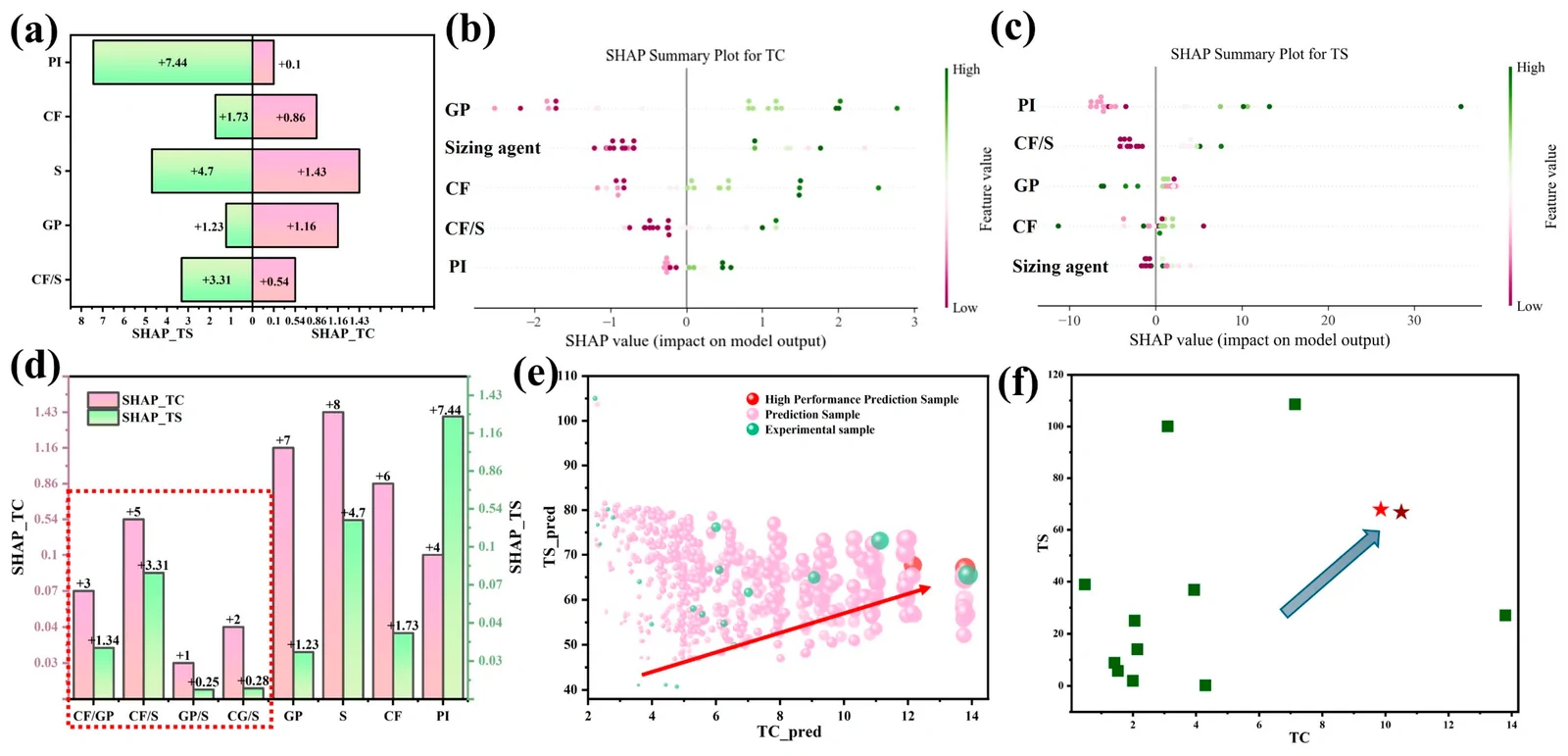
(**a**) Importance ranking of SHAP nested XGBoost algorithm optimal subset. (**b**) Positive/negative correlation of TC and features. (**c**) Positive/negative correlation of TS and features. (**d**) SHAP value of the output of the 8 descriptors of the SHAP nested XGBoost algorithm for Model-Co. (**e**) Scatter plot of predicted TS and TC values for 1957 high-throughput combinations. (**f**) Comparison of mechanical properties and thermal conductivity between this work and other studies [55,56,57,58,59,60,61,62,63,64].

To further explore the knowledge gained from model-Co-TS and model-Co-TC, and to quantify the contribution of each variable to the predictions of TC and TS, SHAP was employed as the model interpretation method. Figure 5a shows the feature importance rankings for the two targets. TS is primarily governed by PI and SA, while TC mainly depends on the synergistic effect of SA and GP. The PI matrix has a negligible impact on TC, whereas CF/SA exhibits a relatively balanced influence on both TS and TC. Table S13, Figure 5b,c illustrate the SHAP dependence of each feature on the two target properties.

The correlation plots between feature values and SHAP values in Figure S12a indicate that increasing the matrix content significantly enhances TS, while a low matrix/high filler ratio is more favorable for improving TC. This phenomenon highlights the importance of balancing mechanical and thermal properties in composite material design. Regarding the role of fillers, Figure S12b,c illustrate that both CF and GP exhibit positive correlations with TC and negative correlations with TS, with the two trends intersecting around 10 wt%. This intersection reflects a trade-off between thermal and mechanical performance. Increasing filler content enhances TC through the formation of conductive networks but simultaneously degrades TS due to agglomeration-induced stress concentration, interfacial debonding, and increased interfacial thermal resistance [1,65]. Incorporating highly thermally conductive fillers, such as carbon materials, is an effective strategy for enhancing the TC of polymers [66,67,68]. This aligns with the general understanding that CF and GP play a key role in this process. As a continuous, load-bearing phase with pronounced anisotropy, CF endows composites with high TS and a high heat deflection temperature [69] while forming efficient thermal conduction pathways along the fiber axis [70]. Adding a small amount of GP to polymers can enhance the overall properties of the composite material due to graphene’s large surface area and multilayered structure [71]. However, single-filler systems are often constrained by factors such as size effects [72] and may struggle to meet multifunctional performance requirements, underscoring the rationale for developing hybrid filler systems.

However, the interface between rigid filler particles and the relatively soft polymer matrix is often a weak point [68]. Under loading, stress tends to concentrate at these interfaces, triggering crack initiation or debonding and causing failure at relatively low stress levels. A high filler content markedly reduces the fraction of the load-bearing continuous polymer matrix, while particulate fillers are prone to agglomeration, forming larger defects [65] that further intensify stress concentration. In this context, SA plays a buffering role. As shown in Figure S12d, despite its small quantity, SA exhibits a pronounced positive correlation with both properties. SA enables bidirectional optimization; its primary role is to maximize strength by improving interfacial adhesion, while it can also enhance thermal conductivity by reducing interfacial thermal resistance. Nevertheless, it cannot fully offset the matrix-weakening effect induced by increasing filler loading (i.e., the decrease in strength with excessive GP remains a clear trend). Compared with CF/GP, GP/SA, and CG/SA (Figure 5d), CF/SA is a more informative indicator of interfacial effects. CF/SA reflects the number of CF interfaces per unit amount of SA and thus characterizes the coverage and wetting capability of SA on the carbon-fiber surface [73]. During the sizing process, SA primarily undergoes chemical interactions with oxygen-containing functional groups on the carbon surface [1], leading to robust adhesion on CFs. The remaining fraction may be physically adsorbed onto CFs. The industry-standard [74] SA content for carbon fibers is typically 0.5–1.5 wt% of fiber mass, corresponding to a CF/SA range of approximately 67–200. In our composite formulations, SA content is defined relative to the total matrix (PI and SA), yielding a much lower CF/SA ratio (typically 0–10). A CF/SA ratio of 2 serves as a critical threshold: below 2, excess SA forms a thick coating that disrupts heat-transfer pathways; above 2, the SA forms a thinner, continuous interfacial layer. Figure S12e shows that when CF/SA exceeds 2, both TS and TC benefit. From an interfacial mechanics perspective, a low CF/SA implies an excess of SA, which tends to form a relatively thick coating layer that disrupts the continuity of heat-transfer pathways [1]. As CF/SA increases, a thinner yet continuous interfacial layer is formed, which strengthens interfacial bonding and improves stress-transfer efficiency [1], thereby promoting concurrent improvements in TS and TC.

By leveraging Model-Co-TS and Model-Co-TC to balance the trade-off between TC and TS, we have identified a dual-optimal solution within the composite formulation space. Figure 5e and Figure S13 present the rapid dual-property prediction landscape for 1957 high-throughput composition candidates. Using the best-performing point in the original dataset (green) as a reference, two high-performance candidates that satisfy the predefined dual-property thresholds were screened out. Part of the Pareto front screening results are shown in Table S14. As shown by the comparison between experimental and predicted results in Table 2, while there are differences in the absolute values, the model accurately captures the overall trends. Given the limited number of validation samples, relative error rather than RMSE was used to evaluate prediction accuracy. The TS prediction error is below 1%, indicating high accuracy. In contrast, the TC error is larger (~20–30%), but it still enables reliable trend prediction and efficient screening. This discrepancy is attributable to the strong sensitivity of thermal conductivity to filler-network formation and interfacial thermal resistance [75,76], microstructural features that are not fully captured by nominal composition inputs. TS, by contrast, is governed mainly by the continuous matrix and is more tolerant of local morphological variations. Consequently, while the model reliably identifies high-performance regions and captures composition-property trends, the absolute prediction of TC remains more challenging. This is an effect commonly observed in composite systems. Compared with the original dataset, where most TC values cluster in the range of 2–6 W·m^−1^·K^−1^, the model-guided validation samples reach approximately 10 W·m^−1^·K^−1^, substantially exceeding the dataset average. The two validated formulations both feature a CF/SA ratio of 2.6. This consistency across two independent samples confirms that CF/SA > 2 serves as a reliable design criterion for identifying formulations with a balanced TS-TC performance. Moreover, Figure 5f benchmarks the TC and TS of our materials against those reported over the past five years [55,56,57,58,59,60,61,62,63,64]. The results show that the validated composites in this work achieve an attractive combination of TC and TS relative to prior studies, indicating that the model can effectively steer the search space toward high-performance regions and uncover promising formulations beyond mere property prediction.

## 4. Conclusions

This work proposes an “experiment-prediction-validation” framework for two-stage ML-driven optimization of CF/PI composites, achieving balanced TS and TC through two-stage optimization. In Stage I, an experimental dataset was used to build a CatBoost model by incorporating polynomial features. Bayesian-optimization-based inverse design indicates that a higher temperature (415–425 °C) and a moderate holding time (30–50 min) are crucial for maximizing TS. In Stage II, a dual-objective XGBoost model was developed by integrating compositional features with the CF/SA ratio as a quantitative descriptor of the interfacial transition layer, leading to a marked improvement in predictive performance for both TS and TC. Interpretability analysis shows that CF and GP contribute positively to TC but negatively to TS, while the CF/SA feature is highly influential for both properties. Notably, formulations with CF/SA > 2 show a tendency toward more favorable TS-TC balance, highlighting the potential role of interfacial engineering in managing mechanical and thermal trade-offs. While the CF/SA ratio is a simplified proxy for complex interfacial physics, it proves practically useful for small-data-driven high-throughput screening. Guided by high-throughput screening and multi-objective selection, experimental validation confirms that the selected composites exhibit a superior thermo-mechanical combination compared with the average level of the original dataset. We acknowledge that the composite dataset used in this study is relatively small (20 samples), and the limited number of experimental validation points (two formulations) means that the CF/SA > 2 trend, while consistent, should be interpreted as a preliminary design guideline rather than a broadly established rule. With additional experimental data, the predictive accuracy and generalizability of the models could be further improved through transfer learning or data augmentation in future work. Nonetheless, this hierarchical framework offers a promising route for rational design of high-performance polymer composites under multi-parameter constraints.

## Figures and Tables

**Table 1:** The inverse design inputs the expected target value, the model gives the feature parameters, and the experiment verifies the target value. Errors in inverse design output values and experimental values.

No.	TS^input^ (MPa)	Pressure^output^ (MPa)	Time^output^ (Min)	T^output^ (℃)	TS^output^ (MPa)	TS^exp^ (MPa)	Δ (MPa)	Δ/TS^exp^ (%)	RMSE
a	80	11.9	40.3	411.4	80.16	86.53	+6.37	7.36	3.89
b	90	18.4	38.2	422.6	90.05	91.34	+1.29	1.41	
c	100	20.0	38.6	419.0	101.26	104.72	+3.46	3.30	
d	110	19.9	39.7	419.4	104.78	102.25	−2.53	2.47	

**Table 2:** PI films with different filler concentrations, as well as values predicted by the models and experimental values.

No.	e	g
CF-content/%	13	13
GP-content/%	11	9
PI-content/%	76	78
SA-content/%	5	5
TS^pred^/MPa	67.02	67.68
TS^exp^/MPa	66.79	67.82
Relative Error	0.34%	0.21%
TC^pred^/W·m^−1^·K^−1^	13.79	12.15
TC^exp^/W·m^−1^·K^−1^	10.50	9.85
Relative Error	31.5%	23.3%

## Data Availability

The original contributions presented in this study are included in the article/Supplementary Material. Further inquiries can be directed to the corresponding authors.

## Supplementary Materials

The following supporting information can be downloaded at: https://www.mdpi.com/article/10.3390/ma19173667/s1, Figure S1: (a) Schematic of the sequential processing route for fabricating carbon-filler/PI composites, illustrating chemical crosslinking and physical adsorption. (b) Mechanistic schematic of the interfacial structure between PI and CF; Figure S2: (a) heatmap of processing parameters and TS. (b) The importance of ranking of feature of the SHAP nested CatBoost algorithm; Figure S3: Box plots and normal distribution plots of experimental TS values of PI films: (a) after outlier removal (thickness range: 10–180 μm); (b) parallel coordinate plot after outlier removal; (c) after thickness filtering; (d) parallel coordinate plot after thickness filtering; Figure S4: Convergence plot of four experimental samples; Figure S5: Prediction Distribution; Figure S6: (a–d) Parameter importance plots for the four candidate targets (80–110 MPa), showing the contribution of each input parameter (time, temperature, pressure) to the predicted performance; Figure S7: 3D Parameter Space; Figure S8: Boxplot and distribution of experimental values of TS and TC of 20 PI-composite films; Figure S9: Scatter plots showing the relationship between TC and TS with filler ratios and SA-content; Figure S10: (a) The changes in R2 and RMSE for model-Co-TS (LOO-CV), built using RFE nested XGBoost with different numbers of descriptors, were accurately described. (b) The importance of ranking of the best-chosen subset of the SHAP nested XGBoost algorithm. Scatter plots of the best model to predict (c) TS and (d) TC values versus their experimental values for the train and test sets; Figure S11: A prediction toolkit for predicting TS and TC of PI films and PI-composites; Figure S12: The relationship between the descriptor values and the SHAP values output of TS and TC for (a) PI-content, (b) CF-content, (c) GP-content, (d) SA-content, and (e) CF/SA; Figure S13: Three-dimensional spatial map of predicted dual performance and evaluation scores for 1957 high-throughput combinations. Pareto frontier of predicted TS versus TC for 1957 high-throughput composition candidates. The red points indicate the two experimentally validated formulations (Table 2). The threshold values (TS ≥ 70 MPa, TC ≥ 12 W·m-1·K-1) used for selecting high-performance candidates. The Pareto-optimal solutions were identified using the NSGA-II algorithm; Table S1: Experimental materials and reagents; Table S2: Experimental instruments and equipment; Table S3: Feature engineering for all PI data; Table S4: Feature engineering of PI data after IQR outlier removal; Table S5: Feature engineering of PI data without thickness feature; Table S6: Different descriptor ordering obtained by polynomial generation-RFE-CatBoost method; Table S7: The optimal hyper-parameters of CatBoost and XGBoost; Table S8: The dataset was randomly divided ten times, trained, and tested by the selected model. To verify the stability and generalization ability of the model-PI, the selected model was used on ten randomly divided train and test sets, the obtained R2 and RMSE, and their average values; Table S9: The model’s reverse mapping outputs and parameters to the process parameter space (* denotes selected experimental samples); Table S10: Feature engineering for composites’ TS (Four basic fillers feature); Table S11: Feature engineering for composites’ TS (Add ratio features); Table S12: Feature engineering for composites’ TC (Add ratio features); Table S13: The influence of four features on two performance; Table S14: PI films with different filler volumes, as well as values predicted by the models. Predicting the outcome of the Pareto fronts: ‘Best Advantage’ and ‘Predicted Scores’ (scores are TC*TS); [77,78,79,80,81,82].
