# Supplementary material for: Two-Stage Machine Learning for Thermo-Mechanical Properties Optimization of Carbon-Filled Polyimide Composites
Source: Materials (Basel). 2026 Aug 28;19(17):3667. doi: 10.3390/ma19173667 (PMC13567010; doi:10.3390/ma19173667)
Supplement: Supplementary file 1 [file materials-19-03667-s001.zip › materials-4472873-supplementary.pdf]

Supplementary Information

Two-Stage Machine Learning for Thermo-Mechanical Properties Optimization of Carbon-Filled Polyimide Composites

Yu Zhang[1], Wenting Zhao[1], Luling He[1], Qiong Li[1], Weibin Ma[1], Tongle Xu[1,3*], Peng Ding[1, 2*]

[1] Research Center of Nano Science and Technology, College of Sciences, Shanghai University, Shanghai200444, P. R. China

[2] Shanghai Key Laboratory of Atomic Control and Application of Inorganic 2D Supermaterials, Shanghai University, 99 Shangda Road, Shanghai 200444, P. R. China

[3]Department of Physics, College of Sciences, Shanghai University, 99 Shangda Road, Shanghai 200444, P. R. China

*Co-Corresponding author: Tongle Xu: xutongle@shu.edu.cn. Peng Ding, Email: dingpeng@shu.edu.cn;

Section S1. General Information

S1. Experimental Procedure

S1.1. Preparation of Carbon Filler

Carbon Fiber (CF) powder was immersed in acetone and sonicated at 60 °C for 2 hours to clean the fiber surface. It was then washed with ethanol until acetone was completely removed and dried at 100°C. The cleaned CF underwent oxidation treatment. The acid oxidation process employed a 3:1 (v/v) concentrated H_2SO_4/HNO_3 mixture with ultrasonic treatment for 30 min. The carbon fibers were placed in an ultrasonic water bath at an operating power of 200 W and a frequency of 53 kHz. The treated CFs were then washed with deionized water to pH 7 and dried, and acidified CFs were obtained. A specific quantity of CFs was dispersed in the prepared GP solution to achieve a mass ratio of 1:1. The mixture was stirred for 60 minutes to form a homogeneous suspension, followed by ultrasonic agitation to uniformly blend the components, yielding the carbon filler GC.

S1.2. Preparation of PSGC Composites

The sizing agent was directly diluted with deionized water to reduce its concentration from 8 wt% to 1 wt%, 2 wt%, 3 wt%, 4 wt%, and 5 wt%, respectively. The carbon filler (GC) was then thoroughly mixed and immersed in the diluted sizing-agent (SA) solution, followed by ultrasonication to ensure homogeneous dispersion. During this process, the reactive epoxy groups in the sizing agent chemically react with the oxygen-containing functional groups (e.g., -OH, -COOH) on CF surface, forming covalent bonds [1]. This creates a chemically bonded interphase layer that strongly adheres to the filler. Excess SA molecules physically adsorb onto this layer, establishing a dual-layer interfacial structure.

After mixing, the suspension was vacuum-filtered to remove excess solvent. The carbon filler was subsequently heated at 150 °C, 200 °C, or 250 °C for 60 min with a ramp rate of 10 °C/min to obtain sized carbon filler (SGC). The resulting samples were denoted as SGC-x (x = 1, 2, 3, 4, 5) according to the sizing-agent concentration. Finally, PI was separately blended with GC and SGC, and the mixtures were hot-pressed (420 °C, 20 MPa, 40 min) to produce the thermoplastic polyimide composites, denoted as PSGC.

S1.3. Definition and content range of SA

SA is a thin polymeric coating applied to CF surfaces to improve processability, protect fibers from abrasion, and enhance interfacial adhesion with the matrix resin [2]. In industrial practice, the SA content on carbon fibers typically ranges from 0.1 to 5 wt% relative to fiber mass, as specified in patents such as JP3807062B2 and EP 3208376 A1.

In this work, SA content is defined relative to the total matrix mass (PI + SA), not relative to CF mass. Given that the CF content in our composites ranges from 10 to 13.3 wt%, a nominal SA content of 5 wt% (relative to matrix) corresponds to an effective SA/CF ratio of approximately 0.5–0.8 wt% when normalized against CF mass, which falls well within the typical industrial range of 0.1–5 wt%. Therefore, 5 wt% SA is entirely appropriate and remains within the definition of a SA.

Relative Error Calculation Formula

$$Relative\ Error = \left|\frac{Predictive\ value - Experimental\ value}{Experimental\ value}\right|$$

S2. Machine learning methods

S2.1 CatBoost (Categorical Boosting)

CatBoost is a gradient boosting algorithm developed by Prokhorenkova [3], specifically designed to handle categorical features efficiently. Unlike traditional gradient boosting methods that require manual encoding of categorical variables (e.g., one-hot encoding), CatBoost employs an innovative strategy called "ordered boosting" and "symmetric trees" to process categorical features directly. The key innovations include the following:

Ordered boosting: A permutation-driven training scheme that avoids target leakage and reduces overfitting by using only historical samples to compute gradients.

Symmetric trees: CatBoost constructs balanced trees where the same splitting criterion is applied across all nodes at the same depth, which speeds up inference and reduces overfitting.

Efficient categorical feature handling: CatBoost converts categorical features into numerical features using target statistics (mean target value per category) with smoothing to prevent overfitting.

These features make CatBoost particularly suitable for small-to-medium datasets with mixed data types, such as our pure PI dataset (140 samples) containing both numerical (temperature, pressure, time) and derived interaction features.

S2.2 XGBoost (eXtreme Gradient Boosting)

XGBoost [4] is a scalable tree boosting system that has become one of the most widely used ML algorithms for tabular data. It builds an ensemble of decision trees sequentially, where each new tree attempts to correct the errors of the previous trees. The key features of XGBoost include the following:

Regularized objective function: Unlike traditional gradient boosting that minimizes only the training loss, XGBoost includes both L1 (lasso) and L2 (ridge) regularization terms to penalize model complexity, effectively controlling overfitting.

Second-order gradient approximation: XGBoost uses both first and second derivatives of the loss function (Taylor expansion), which provides a more accurate direction for tree construction compared to using only first derivatives.

Sparsity-aware split finding: XGBoost can handle missing values and sparse features by learning default directions for missing values.

Tree pruning with max_depth: Unlike traditional boosting that continues growing trees until a stopping criterion is met, XGBoost grows trees depth-first and prunes them backward to remove splits that do not contribute to performance gain.

In this study, XGBoost was chosen for Stage II composite formulation modeling due to its robustness against overfitting (via regularization) and strong performance on small tabular datasets (20 samples).

S2.3 Bayesian Optimization with Gaussian Process

Bayesian optimization [5] is an efficient global optimization technique for expensive black-box functions. Unlike grid search or random search, Bayesian optimization builds a probabilistic surrogate model (typically a Gaussian Process) to approximate the true objective function, then uses an acquisition function to decide where to sample next. The algorithm proceeds as follows:

Surrogate model: A Gaussian Process (GP) defines a prior distribution over possible objective functions. The GP is fully specified by its mean function *m(x)* and covariance (kernel) function $k(x, x')$, which encodes assumptions about smoothness and correlation between points.

Acquisition function: After updating the GP with observed data, an acquisition function (e.g., Expected Improvement, Upper Confidence Bound) balances exploration (sampling where uncertainty is high) and exploitation (sampling where the predicted mean is high). The point that maximizes the acquisition function is selected for the next evaluation.

Iterative update: The objective function is evaluated at the selected point, the GP is updated with the new observation, and the process repeats until convergence.

In this work, we applied Bayesian optimization to inversely design hot-pressing parameters (temperature, pressure, time) for target tensile strength values. The objective function was defined as the squared error between the model-predicted TS and the target TS. After 200 iterations, the optimal parameter sets corresponding to the minimum objective values were identified.

S2.4 SHAP (SHapley Additive exPlanations)

SHAP[6] is a unified framework for interpreting model predictions based on cooperative game theory. It assigns each feature an importance value (SHAP value) representing its contribution to the prediction difference from the average. The key principles are as follows:

Shapley values: Originating from cooperative game theory, Shapley values fairly distribute the total payoff among players (features) based on their marginal contributions across all possible coalitions. The SHAP value for feature *i* is the average marginal contribution of that feature over all possible feature subsets.

Additive feature attribution: SHAP explains a prediction as a linear combination of binary variables indicating feature presence:

$$g(z') = \emptyset_0 + \sum_{i=1}^{M} \emptyset_i z'_i$$

where $\emptyset_i$ is the SHAP value for feature ii, and z'_i indicates whether feature *i* is observed or missing.

Local and global interpretability: SHAP provides both local explanations (why a single prediction has a certain value) and global explanations (average feature importance across all predictions). The direction (positive or negative) of a feature's contribution is directly interpretable from the sign of the SHAP value.

Model-agnostic: SHAP can be applied to any machine learning model, including tree-based models (CatBoost and XGBoost) via efficient approximation algorithms such as TreeExplainer.

In this study, SHAP was used to interpret both the CatBoost model (Stage I) and the XGBoost models (Stage II), revealing the contributions of processing parameters and formulation variables to tensile strength and thermal conductivity. The SHAP summary plots (Figures 6 and 10) illustrate which features drive predictions and whether their effects are positive or negative.

S2.5 High-throughput screening and multi-objective selection

Python implementation of the NSGA-II-based Pareto optimization for multi-objective screening of CF/PI composite formulations. The code loads the trained XGBoost models (model-Co-TS and model-Co-TC), generates 1,957 composition candidates, and identifies the Pareto-optimal solutions.

Supplementary Tables

Table S1. Experimental materials and reagents.

Raw material	Specification	Source
Thermoplastic PI	PD450	Tianjin Geqite New Materials
CF	TYMF	Hunan Dongying Carbon Materials Technology Co., Ltd.
Graphene (GP, KNG-180-3)	98.8%	Xiamen Kaina Graphene Technology Co., Ltd.
H_2SO_4	Analytical grade	Sinopharm Chemical Reagent Co., Ltd.
HNO_3	Analytical grade	Sinopharm Chemical Reagent Co., Ltd.
Acetone	99.5%	China Fortune Chemical Reagent Co., Ltd.
Sizing agent(SA)	TX-SAI-S01	Ningbo Institute of Materials Technology and Engineering, Chinese Academy of Sciences (NIMTE, CAS)

Table S2. Experimental instruments and equipment.

Instrument	Model	Manufacturer
Electronic analytical balance	FA2204C	Shanghai Jingke Tianmei Scientific Instruments
Ultrasonic cleaner	SK5210SHC	Shanghai Kedao Ultrasonic Instruments
Vacuum drying oven	DZF-6050	Shanghai Jinghong Laboratory Equipment Co., Ltd.
Heavy-duty electric overhead stirrer	JB200-D	Shanghai Biaoben Model Factory
Pneumatic flat-plate vulcanizing press	BD-8820-F-30T	Dongguan Baoding Precision Instruments Co., Ltd.
Muffle furnace	KYS-1200C	Hefei Microelement Laboratory Equipment Co., Ltd.

Table S3. Feature engineering for all PI data.

feature engineering	algorithm	Train		Test	
		R^2	RMSE	R^2	RMSE
polynomial generation + RFE	Ridge	0.41	12.71	0.45	11.10
	Lasso	0.38	13.11	0.38	11.81
	CatBoost*	0.93	4.43	0.75	7.51
	PLS	0.15	15.37	0.16	13.70
	ElasticNet	0.37	13.18	0.41	11.53
featuretools	Ridge	0.30	13.90	0.31	12.40
autofeat	Ridge	0.37	13.16	0.37	11.84
	DT (rule)	0.43	11.31	0.28	17.61
	XGBoost (rule)	0.85	6.25	0.74	7.78
	symbolic regression (genetic algorithm)	0.18	14.38	0.09	17.04
	symbolic regression (neural network)	0.19	14.89	0.08	14.72

*[“temperature pressure,” “temperature time,” “pressure time,” “pressure thickness,” “time^2”].

Table S4. Feature engineering of PI data after IQR outlier removal.

feature engineering	algorithm	Train		Test	
		R^2	RMSE	R^2	RMSE
	Ridge	0.51	11.02	0.50	10.92

	Lasso	0.52	11.17	0.47	10.97
polynomial generation + RFE	CatBoost*	0.92	4.57	0.76	7.84
	PLS	0.26	13.94	0.25	12.61
	ElasticNet	0.49	11.60	0.49	10.37
featuretools	Ridge	0.40	12.33	0.45	13.58
autofeat	Ridge	0.44	11.88	0.46	11.71
	DT	0.54	10.95	0.51	10.22
	XGB	0.85	6.17	0.70	8.71
	symbolic regression (genetic algorithm)	0.27	14.12	0.30	10.51
	symbolic regression (neural network)	0.27	13.84	0.26	12.56

* [“temperature,” “time,” “temperature pressure,” “temperature time,” “pressure thickness,” “time^2,” “time thickness”]

Table S5. Feature engineering of PI data without thickness feature.

feature engineering	algorithm	Train		Test	
		R^2	RMSE	R^2	RMSE
	Ridge	0.64	6.93	0.67	6.64
	Lasso	0.59	7.37	0.62	7.15
polynomial generation + RFE	CatBoost*	0.9997	0.22	0.97	1.99
	PLS	0.36	9.80	0.37	7.22
	ElasticNet	0.53	7.94	0.59	7.48
featuretools	Ridge	0.54	8.21	0.55	6.09
autofeat	Ridge	0.54	6.84	0.50	11.67
	DT	0.73	6.15	0.73	5.24
	XGB	0.98	1.65	0.96	2.29
	symbolic regression (genetic algorithm)	0.57	7.58	0.59	7.19
	symbolic regression (neural network)	0.37	7.90	0.34	13.47

* [“time,” “temperature,” “time temperature,” “temperature^2,” “temperature pressure”]

Table S6. Different descriptor ordering obtained by polynomial generation-RFE-CatBoost method.

Number	Model-PI
1	['time']
2	['time', 'temperature']
3	['time', 'temperature', 'time temperature']
4	['time', 'temperature', 'time temperature', 'temperature^2']
5	*['time', 'temperature', 'time temperature', 'temperature^2', 'temperature pressure']
6	['time', 'temperature', 'time^2', 'time temperature', 'temperature^2', 'temperature pressure']
7	['time', 'temperature', 'time^2', 'time temperature', 'time pressure', 'temperature^2', 'temperature pressure']
8	['time', 'temperature', 'time^2', 'time temperature', 'time pressure', 'temperature^2', 'temperature pressure', 'pressure^2']
9	['time', 'temperature', 'pressure', 'time^2', 'time temperature', 'time pressure', 'temperature^2', 'temperature pressure', 'pressure^2']

Table S7. The optimal hyper-parameters of CatBoost and XGBoost.

hyper-parameter	CatBoost (PI) Value	XGBoost (Co-TS) Value	XGBoost (Co-TC) Value
Learning rate	0.3	0.1	0.2
l2_leaf_reg	10	-	-
max depth	4	6	3
subsample	0.9	0.7	0.9
iterations	400	-	
n_estimators	-	100	100

reg_lambda	-	1	3
colsample_bytree	-	0.8	0.8

Table S8. The dataset was randomly divided ten times, trained, and tested by the selected model. To verify the stability and generalization ability of the model-PI, the selected model was used on ten randomly divided train and test sets, the obtained R^2 and RMSE, and their average values.

	**Train**		**Test**	
	RMSE	R^2	RMSE	R^2
1	0.21	0.999717	2.86	0.90
2	0.25	0.999596	3.25	0.71
3	0.21	0.999689	3.54	0.90
4	0.21	0.999706	2.39	0.94
5	0.20	0.999689	3.20	0.92
6	0.18	0.999783	2.91	0.90
7	0.22	0.999638	2.37	0.96
8	0.29	0.999336	3.79	0.91
9	0.29	0.999416	3.01	0.88
10	0.26	0.999489	2.78	0.95
mean	0.23	0.999606	3.01	0.90

Table S9. The model's reverse mapping outputs and parameters to the process parameter space (* denotes selected experimental samples).

**Input**	**Parameter space**	**Output**	**Objective value**
80*	time	80.15	0.025
90*	10-90	90.05	0.0029
100	temperature<br>400-430	98.30	2.88
110	pressure<br>10-20	87.42	509.83
80	time	78.27	3.00
90	35- 45	90.16	0.026
100*	temperature<br>415-425	101.26	1.58
110*	pressure<br>15-20	104.78	27.30

Table S10. Feature engineering for composites' TS (Four basic fillers feature).

**feature engineering**	**algorithm**	**Train**		**Test**	
		**R^2**	**RMSE**	**R^2**	**RMSE**
RFE	Ridge	0.08	15.17	0.18	11.47
	XGBoost*	1	0.002	0.92	3.49
polynomial generation + RFE	Ridge	0.25	13.07	0.27	13.09
	CatBoost	0.9998	0.26	0.77	3.94
	ElasticNet	0.02	15.72	-	12.66
featuretools	Ridge	0.71	8.41	0.72	6.72
	DT	0.94	3.34	0.64	8.38
	XGBoost	0.998	0.74	0.85	4.98
	symbolic regression (genetic algorithm)	0.88	4.13	0.51	12.57
	symbolic regression (neural network)	0.89	5.03	0.79	5.80

* ["PI-content," "CF-content," "Sizing agent-content"]

Table S11. Feature engineering for composites' TS (add ratio features).

**n_features**	**Selected_Features**

8	PI-content, CF-content, GP-content, Sizing agent-content, CF/GP, CF/S, GP/S, CG/S
7	PI-content, CF-content, GP-content, Sizing agent-content, CF/S, GP/S, CG/S
6	PI-content, CF-content, GP-content, Sizing agent-content, CF/S, CG/S
5*	PI-content, CF-content, GP-content, Sizing agent-content, CF/S
4	PI-content, CF-content, Sizing agent-content, CF/S
3	PI-content, CF-content, CF/S
2	PI-content, CF/S
1	PI-content

Table S12. Feature engineering for composites' TC (add ratio features).

n_features	Selected_Features
8	PI-content, CF-content, GP-content, Sizing agent-content, CF/GP, CF/S, GP/S, CG/S
7	PI-content, CF-content, GP-content, Sizing agent-content, CF/S, GP/S, CG/S
6	PI-content, CF-content, GP-content, Sizing agent-content, CF/S, CG/S
5*	PI-content, CF-content, GP-content, Sizing agent-content, CF/S
4	CF-content, GP-content, Sizing agent-content, CF/S
3	CF-content, GP-content, CF/S
2	CF-content, GP-content
1	PI-content

Table S13. PI films with different filler volumes, as well as values predicted by the models. Predicting the outcome of the Pareto fronts: "Best Advantage" and "Predicted Scores" (scores are TC*TS).

PI	CF	S	GP	CF/S	TC^{pred}	TS^{pred}	Pareto	Score
0.75	0.13	0.05	0.12	2.60	13.79	67.02	TRUE	924.25
0.75	0.135	0.05	0.115	2.70	13.77	64.62	FALSE	889.62
0.75	0.14	0.05	0.11	2.80	13.77	64.62	FALSE	889.62
0.75	0.145	0.05	0.105	2. 90	13.77	64.62	FALSE	889.62
0.775	0.11	0.0214	0.115	5.14	11.98	73.46	TRUE	879.72
0.775	0.115	0.0214	0.11	5.37	11.98	73.46	TRUE	879.72
0.775	0.12	0.0214	0.105	5.61	11.98	73.46	TRUE	879.72
0.75	0.13	0.0214	0.12	6.07	11.93	73.38	FALSE	875.38
0.75	0.13	0.025	0.12	5.20	11.93	73.38	FALSE	875.38
0.775	0.125	0.0214	0.1	5.84	11.93	73.38	FALSE	875.38
0.75	0.135	0.0214	0.115	6.31	11.92	70.62	FALSE	841.61
0.75	0.135	0.025	0.115	5.40	11.92	70.62	FALSE	841.61
0.75	0.14	0.0214	0.11	6.54	11.92	70.62	FALSE	841.61
0.75	0.14	0.025	0.11	5.60	11.92	70.62	FALSE	841.61
0.75	0.145	0.0214	0.105	6.78	11.92	70.62	FALSE	841.61
0.75	0.145	0.025	0.105	5.80	11.92	70.62	FALSE	841.61
0.75	0.145	0.0286	0.105	5.07	11.92	70.62	FALSE	841.61
0.75	0.12	0.0214	0.13	5.61	11.94	70.15	FALSE	837.94
0.75	0.125	0.0214	0.125	5.84	11.90	70.19	FALSE	835.26
0.775	0.13	0.05	0.095	2.60	12.15	67.68	TRUE	822.59

Table S14. The influence of four features on two performances.

No.	TS	TC
CF-content	-(＜0.02)	+(＞0.10)
GP-content	-(0.1)	+(0.1)
PI-content	+(＞0.85)	＞0.90
Sizing agent-content	+(⩾0.01)	+(＞0.01)

Supplementary Figures

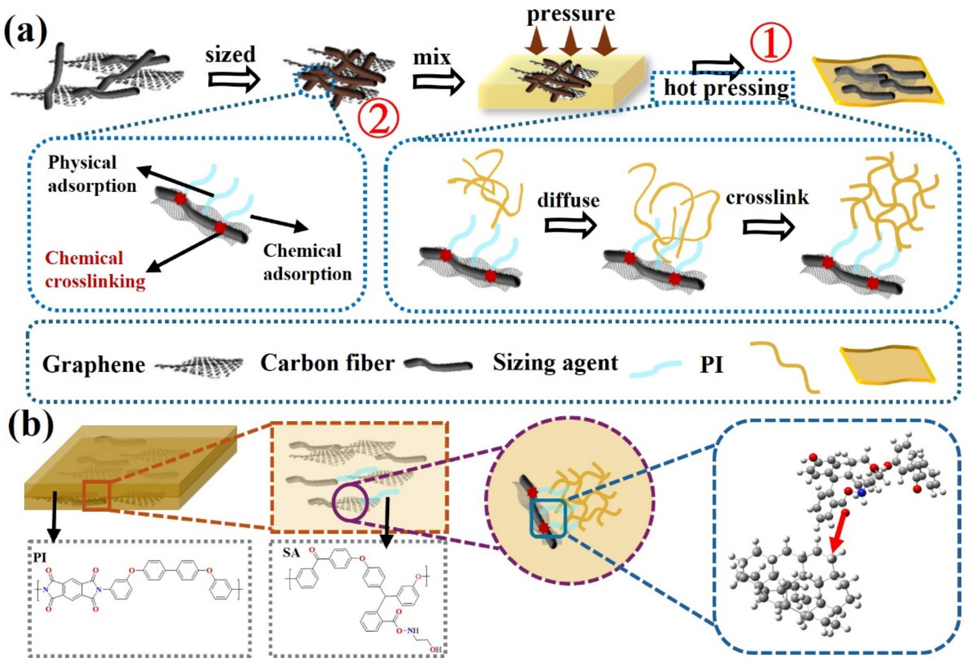

Figure S1. (a) Schematic of the sequential processing route for fabricating carbon-filler/PI composites, illustrating chemical crosslinking and physical adsorption. (b) Mechanistic schematic of the interfacial structure between PI and CF.

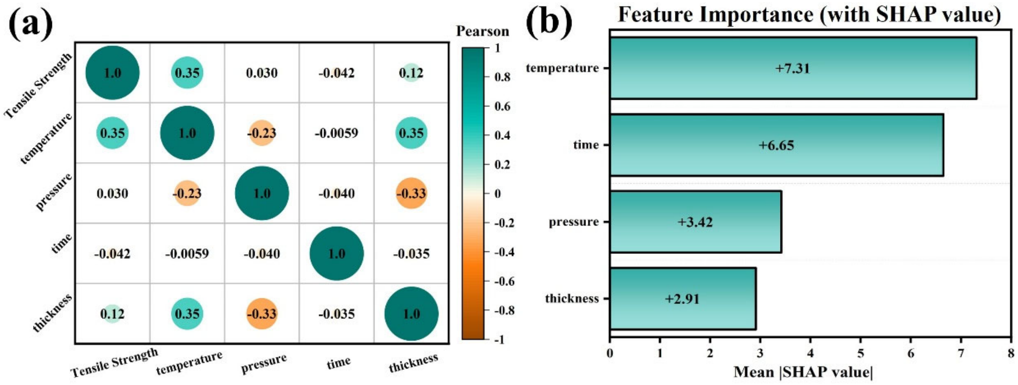

Figure S2. (a) Heatmap of processing parameters and TS. (b) The importance of ranking of feature of the SHAP nested CatBoost algorithm.

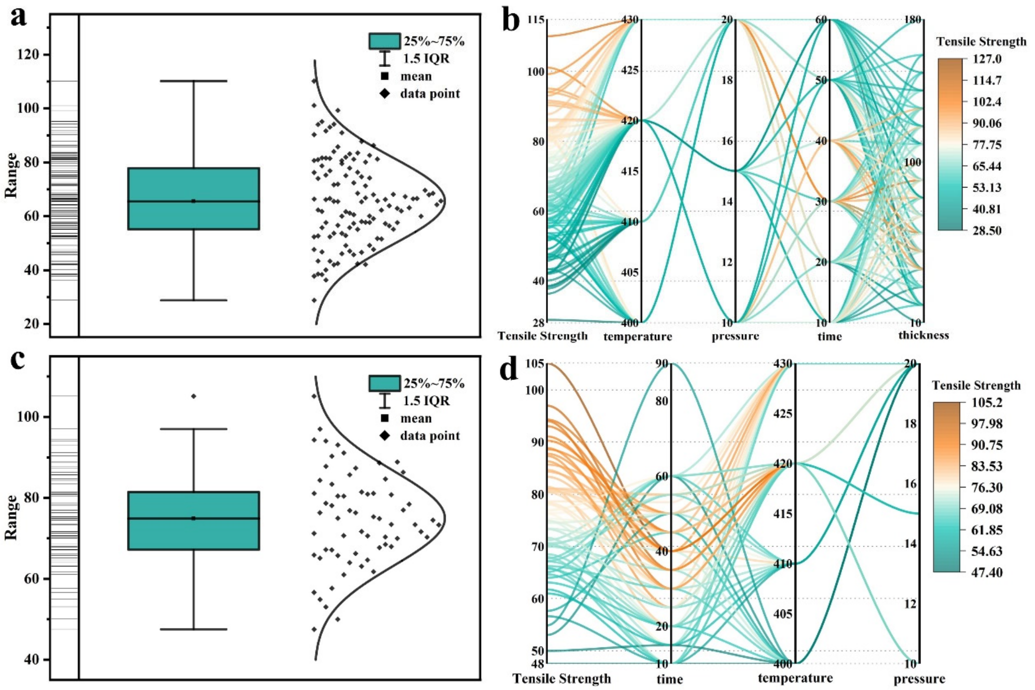

Figure S3. Box plots and normal distribution plots of experimental TS values of PI films: (a) after outlier removal (thickness range: 10-180 μm); (b) parallel coordinate plot after outlier removal; (c) after thickness filtering; (d) parallel coordinate plot after thickness filtering.

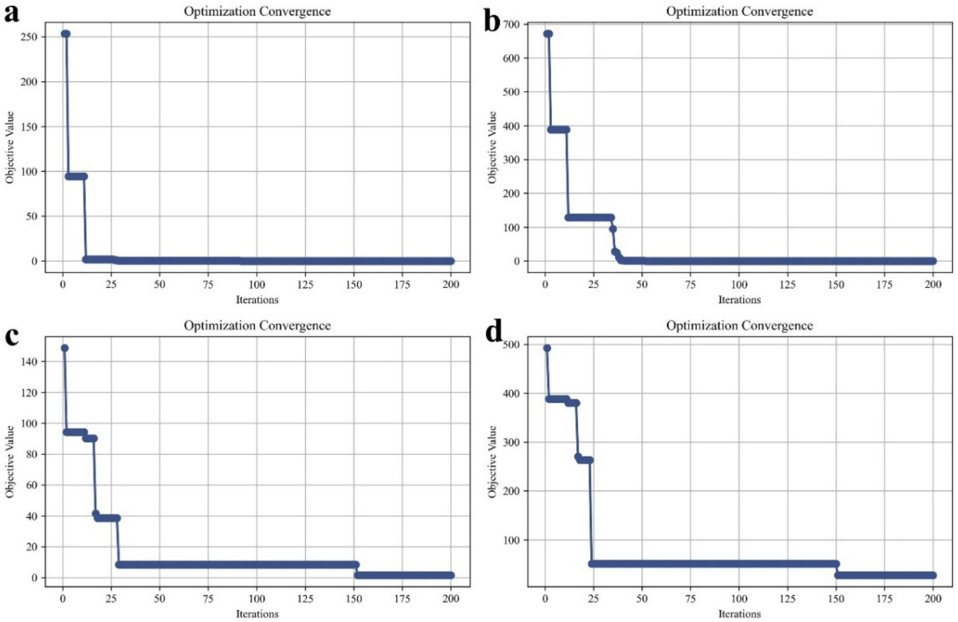

Figure S4. Convergence plot of four experimental samples.

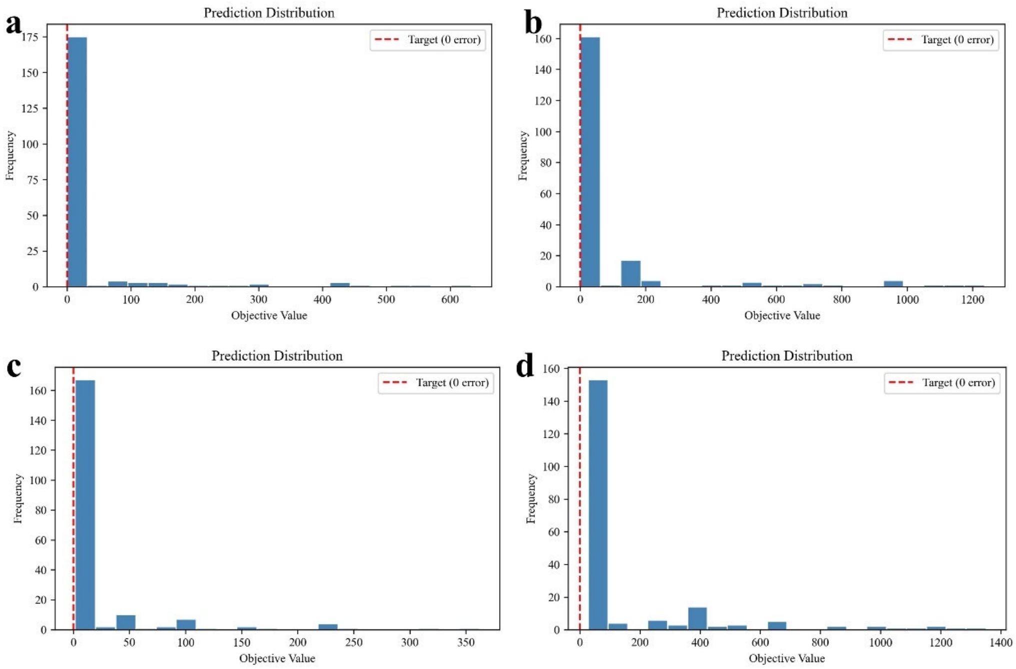

Figure S5. Prediction distribution.

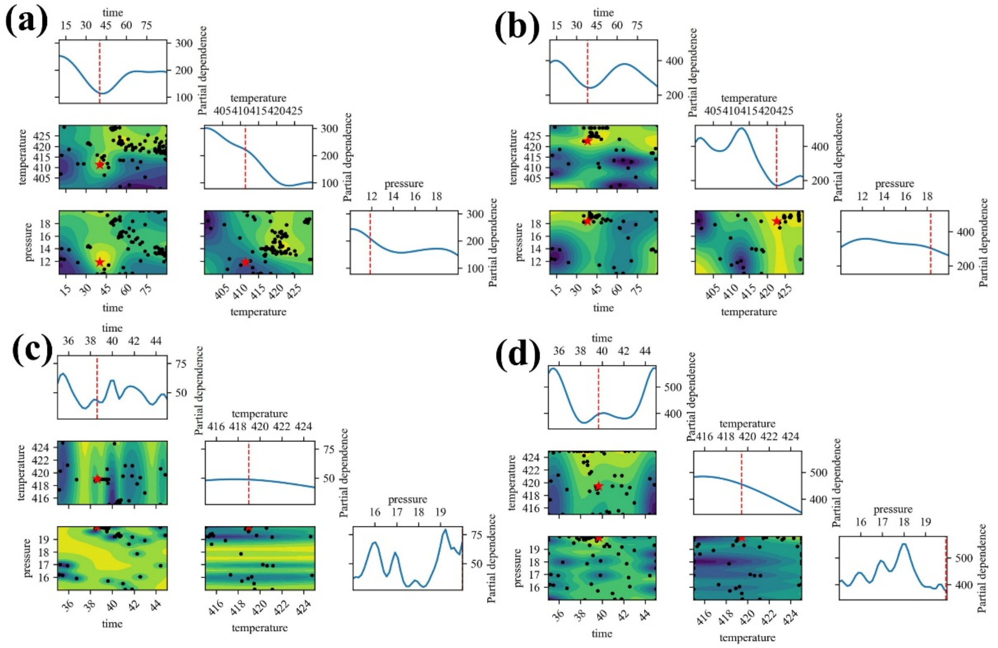

Figure S6. (a-d) Parameter importance plots for the four candidate targets (80–110 MPa), showing the contribution of each input parameter (time, temperature, pressure) to the predicted performance.

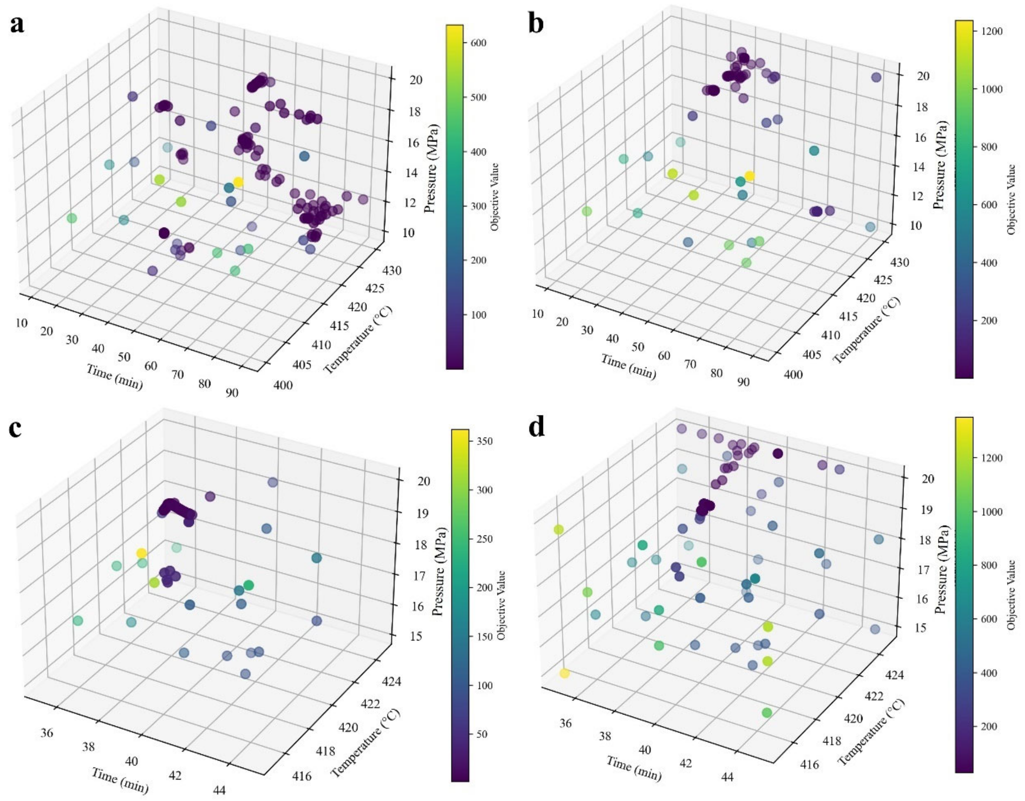

Figure S7. 3D parameter space.

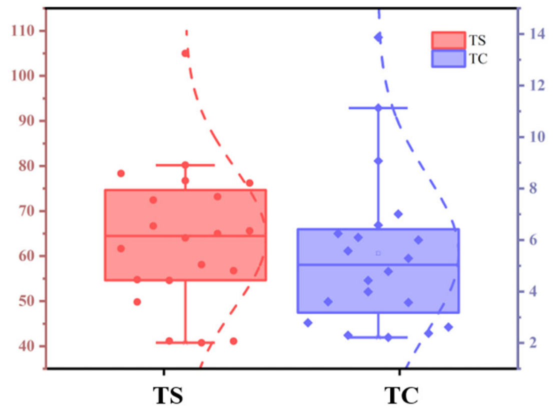

Figure S8. Boxplot and distribution of experimental values of TS and TC of 20 PI-composite films.

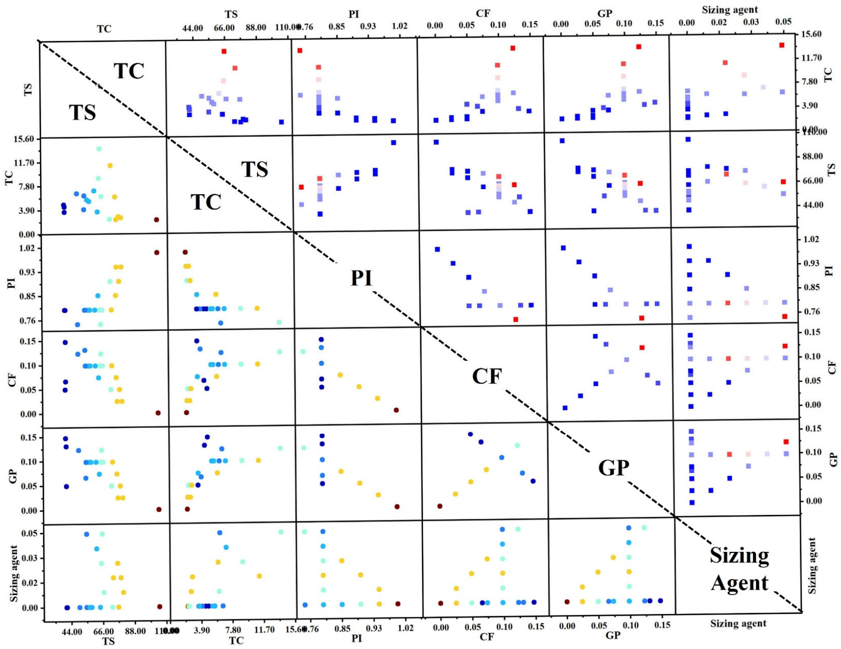

Figure S9. Scatter plots showing the relationship between TC and TS with filler ratios and SA-content.

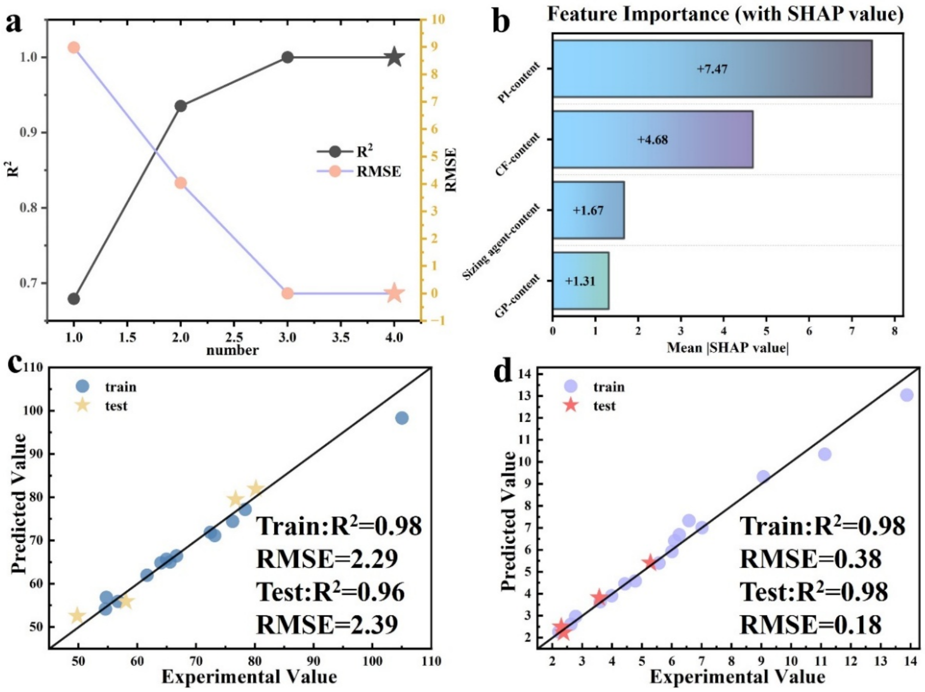

Figure S10. (a) The changes in R^2 and RMSE for model-Co-TS (LOO-CV), built using RFE nested XGBoost with different numbers of descriptors, were accurately described. (b) The importance of ranking of the best-chosen subset of the SHAP nested XGBoost algorithm. Scatter plots of the best model to predict (c) TS and (d) TC values versus their experimental values for the train and test sets.

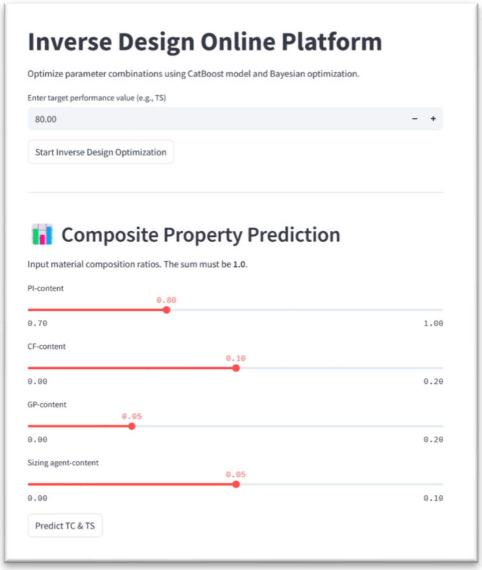

Figure S11. A prediction toolkit for predicting TS and TC of PI films and PI-composites.

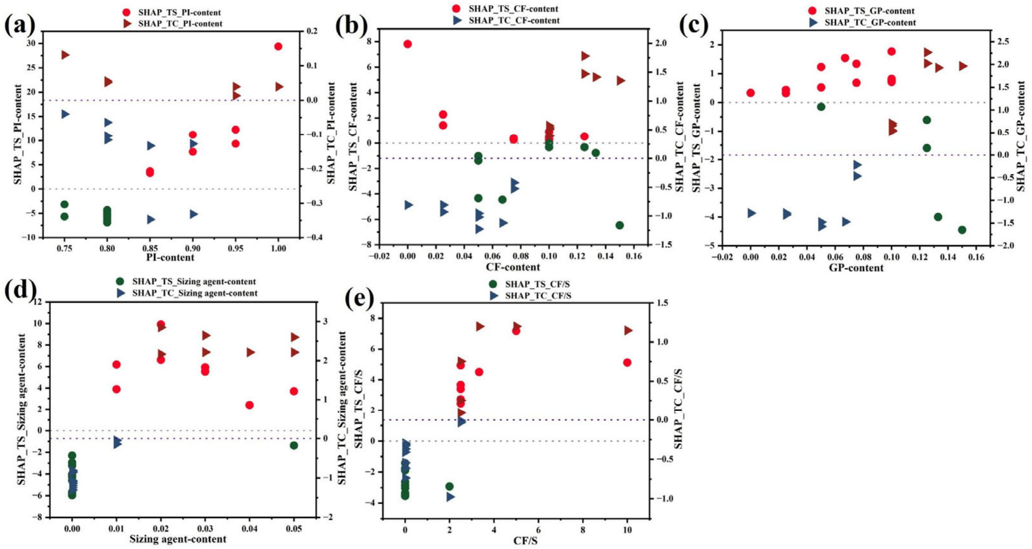

Figure S12. The relationship between the descriptor values and the SHAP values output of TS and TC for (a) PI-content, (b) CF-content, (c) GP-content, (d) SA-content, and (e) CF/SA.

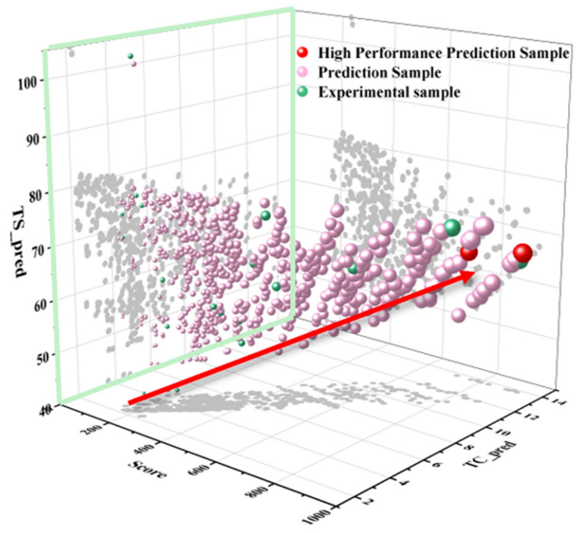

Figure S13. Three-dimensional spatial map of predicted dual performance and evaluation scores for 1,957 high-throughput combinations. Pareto frontier of predicted TS versus TC for 1,957 high-throughput composition candidates. The red points indicate the two experimentally validated formulations (Table 2). The threshold values (TS ≥ 70 MPa, TC ≥ 12 $W{\cdot}m^{-1}{\cdot}K^{-1}$) used for selecting high-performance candidates. The Pareto-optimal solutions were identified using the NSGA-II algorithm.

References

77. Moosburger-Will, J.; Bauer, M.; Laukmanis, E.; Horny, R.; Wetjen, D.; Manske, T.; Schmidt-Stein, F.; Töpker, J.; Horn, S., Interaction between carbon fibers and polymer sizing: Influence of fiber surface chemistry and sizing reactivity. *Applied Surface Science* **2018,** 439, 305-312.
78. Pawar, S. S.; Hutchinson, S. A.; Eyckens, D. J.; Stojcevski, F.; Hayne, D. J.; Gengenbach, T. R.; Razal, J. M.; Henderson, L. C., Carbon fiber sizing agents based on renewable terpenes. *Composites Science and Technology* **2022,** 220, 109280.
79. Prokhorenkova, L.; Gusev, G.; Vorobev, A.; Dorogush, A. V.; Gulin, A. In *CatBoost: unbiased boosting with categorical features*, 32nd Conference on Neural Information Processing Systems (NIPS), Montreal, CANADA, Dec 02-08 2018; Montreal, CANADA, 2018.
80. Chen, T. Q.; Guestrin, C.; Assoc Comp, M. In *XGBoost: A Scalable Tree Boosting System*, 22nd ACM SIGKDD International Conference on Knowledge Discovery and Data Mining (KDD), San Francisco, CA, Aug 13-17 2016; San Francisco, CA, 2016; pp 785-794.
81. Snoek, J.; Larochelle, H.; Adams, R. P., Practical Bayesian Optimization of Machine Learning Algorithms. *Advances in neural information processing systems* **2012,** 4.
82. Lundberg, S. M.; Lee, S. I. In *A Unified Approach to Interpreting Model Predictions*, 31st Annual Conference on Neural Information Processing Systems (NIPS), Long Beach, CA, Dec 04-09 2017; Long Beach, CA, 2017.
